# Transcriptional vulnerabilities of striatal neurons in human and rodent models of Huntington’s disease

**DOI:** 10.1038/s41467-022-35752-x

**Published:** 2023-01-17

**Authors:** Ayano Matsushima, Sergio Sebastian Pineda, Jill R. Crittenden, Hyeseung Lee, Kyriakitsa Galani, Julio Mantero, Geoffrey Tombaugh, Manolis Kellis, Myriam Heiman, Ann M. Graybiel

**Affiliations:** 1 McGovern Institute for Brain Research, Massachusetts Institute of Technology, Cambridge, MA USA; 2 Department of Brain and Cognitive Sciences, Massachusetts Institute of Technology, Cambridge, MA USA; 3 Picower Institute for Learning and Memory, Massachusetts Institute of Technology, Cambridge, MA USA; 4 Broad Institute of MIT and Harvard, Cambridge, MA USA; 5 Department of Electrical Engineering and Computer Science, MIT, Cambridge, MA USA; 6 MIT Computer Science and Artificial Intelligence Laboratory, Cambridge, MA USA; 7 PyschoGenics Inc., Paramus, NJ USA

**Keywords:** Molecular neuroscience, Huntington's disease, Transcriptomics

## Abstract

Striatal projection neurons (SPNs), which progressively degenerate in human patients with Huntington’s disease (HD), are classified along two axes: the canonical direct-indirect pathway division and the striosome-matrix compartmentation. It is well established that the indirect-pathway SPNs are susceptible to neurodegeneration and transcriptomic disturbances, but less is known about how the striosome-matrix axis is compromised in HD in relation to the canonical axis. Here we show, using single-nucleus RNA-sequencing data from male Grade 1 HD patient post-mortem brain samples and male zQ175 and R6/2 mouse models, that the two axes are multiplexed and differentially compromised in HD. In human HD, striosomal indirect-pathway SPNs are the most depleted SPN population. In mouse HD models, the transcriptomic distinctiveness of striosome-matrix SPNs is diminished more than that of direct-indirect pathway SPNs. Furthermore, the loss of striosome-matrix distinction is more prominent within indirect-pathway SPNs. These results open the possibility that the canonical direct-indirect pathway and striosome-matrix compartments are differentially compromised in late and early stages of disease progression, respectively, differentially contributing to the symptoms, thus calling for distinct therapeutic strategies.

## Abstract (web summary)

In human and mouse models of Huntington’s disease, Matsushima, Pineda et al. show, using snRNAsequencing, the two axes defining identities of striatal projection neurons are multiplexed and differentially compromised, calling for distinct therapies.

## Introduction

Huntington’s disease (HD) is a major extrapyramidal disorder typically characterized by early-stage mood and cognitive disorders, a subsequent hyperkinetic followed by hypokinetic ‘manifest’ stage, and an eventual decline to death^1^. Expansion of uninterrupted CAG repeats at the locus of *HTT* gene (*mHTT*) reaching over 40 results in manifest HD. A hallmark of HD is the profound loss of neurons in the neostriatum. Work on zQ175 and R6/2 models and human HD brain samples has documented marked anatomic and electrophysiologic alterations within the striatum. Especially vulnerable are striatal spiny projection neurons (SPNs) expressing D2 dopamine receptors and giving rise to the indirect pathway of the basal ganglia (iSPNs)^2–5^. With time, multiple striatal cell types become affected, including the direct-pathway SPNs expressing D1 dopamine receptors (dSPNs) and even glial cells, leading to cavitation of the striatum. These pathophysiologic patterns are concordant with the hyperkinetic followed by hypokinetic motor symptomatology in HD, as the strongly and first perturbed iSPNs normally support motor inhibition, whereas dSPNs support motor release.

A second, less fully studied feature of striatal vulnerability has been found to involve the neurochemical compartmental organization of the striatum, in which molecularly specialized labyrinthine ‘striosomes’ wind through the surrounding matrix compartment^6^. Both striosomes and matrix contain dSPNs as well as iSPNs^7–10^, and, like the D1-D2 axis of striatal organization, the striosome-matrix (S-M) axis specifies input-output connectivities. The fact that anteromedial striosomes preferentially receive inputs from limbic circuits and much of the matrix does from sensory-motor circuits^11,12^ has raised interest in the possibility that dysregulation along the S-M axis could be related to the modal transition of HD symptomatology over time. Reports of early vulnerability of striosomes based on post-mortem anatomy^13,14^, especially in identified mood-disorder patients^15^, have led to the view that striosomal dysfunction could differentially contribute to the pre-manifest periods, with mood disorders, then merge with following motor dysfunction as the matrix becomes increasingly affected^14,16^.

Transcriptomic studies have since also indicated differential vulnerability of the striosomes and matrix in HD. Substance P/*Tac1*, a marker of striosomes, was found to be downregulated especially in dSPNs, suggestive of an intensified loss of markers of the striosome compartment^17^. However, it is not yet clear how transcriptional dysregulation in SPNs along the S-M organizational axis of the striatum relates to the dysregulation of the seemingly orthogonal D1-D2 (dSPN-iSPN) axis of organization. Here, to address this issue, we leveraged single-nucleus RNA-sequencing (snRNA-seq) to examine striatal transcripts derived from the human HD striatum, and from zQ175 and R6/2 mouse models of HD, the classic R6/2 model with rapid progression, and the knock-in zQ175 model with slower progression. For the human as well as rodent transcripts, we applied advanced sub-clustering of dSPN and iSPN populations and used a curated set of S-M marker genes to annotate sub-clusters corresponding to striosomes and matrix inside them^18,19^. With this base, we then compared the differential transcriptomic changes according to their compartmental sub-clusters and by their D1-D2 parent clusters.

Our evidence demonstrates that, despite the dominant dysregulation of D2-expressing iSPNs as widely accepted, the transcriptomic profiles differentiating striosomal SPNs from matrix SPNs were more disrupted than those distinguishing dSPNs from iSPNs. Transcripts differentiating striosomal and matrix SPNs, classified as S-M ‘markers’ in our analysis, were dysregulated in a cell-type-specific manner so as to blur the endogenous transcriptional distinctiveness of the two compartments. Striosomal SPNs exhibited upregulated matrix markers but downregulated striosome markers, thus diminishing their striosome-like identity. Matrix SPNs, on the other hand, exhibited upregulated striosome markers but downregulated matrix markers, thus diminishing their matrix-like identity. In sharp contrast, D1-D2 marker transcripts were dysregulated irrespective of cell type, largely preserving the distinctiveness between them. Thus, both zQ175 and R6/2 models of HD exhibited a cohesive pattern tending to cancel out the endogenous identities of striosomal and matrix SPNs, whereas the distinction along the D1-D2 (dSPN-iSPN) axis was more robust despite the greater disturbances in iSPNs, as confirmed in the human Grade 1 case.

In both zQ175 and R6/2 mouse models, we also found that the absolute degree of cell-type-specific dysregulation of gene expression, whether up or down, was most prominent in a particular identified set of putative iSPNs that we classified as ‘outlier-D2’ (O-D2), followed by striosomal iSPNs (S-D2). The O-D2 SPNs formed a small sub-cluster within the parent D2 cluster. This pattern accords with the well-known D2-dominant deficits in HD and was consistently observed in dysregulations of all genes including non-markers, meeting the criterion of being cell-type-specific (i.e., dysregulated in opposite directions in different cell types). It was for the genes that were dysregulated in the same directions across cell types that the zQ175 and R6/2 models exhibited unique transcriptomic alterations, likely reflecting their distinct genetic makeups. The severe loss of SPNs in HD patients, which was not found in zQ175 and R6/2 models, hindered in-depth transcriptomic analysis in human samples. Yet, we were able to find two clues that key dysregulation patterns are coherent in human and mouse. First, in a Grade 1 HD case, cell-type-specific vulnerability was congruent with zQ175 and R6/2 HD modes in the pattern of depletions: S-D2 were the most severely depleted SPNs of the entire SPN population, followed by S-D1 and M-D2. Second, transcriptomic distinctiveness along S-M axis were disrupted in the human HD case striosome markers were more downregulated in striosomal SPNs than in matrix SPNs, whereas matrix markers were more downregulated in matrix iSPNs than striosomal iSPNs.

In this study, we suggest that transcriptomic dysregulation in HD is dependent not only on the canonical D1-D2 pathway organization of SPNs, but also on their compartmental striosome-matrix organization. In the human Grade 1 HD brain, striosomal and indirect-pathway SPNs are the most depleted. In the human HD case and in zQ175 and R6/2 models, the loss of transcriptomic distinction between striosome and matrix is more prominent in indirect-pathway SPN populations. This result suggests a decidedly multiplexed order of SPN-type-specific vulnerability in the striatum in HD. We suggest that these patterns could differentially contribute to the pre-manifest and manifest stages of this devastating basal ganglia disorder.

## Results

### Identification of earliest-depleted SPN subtype in human HD and of SPN-subtype-specific markers across species

**Fig. 1:**
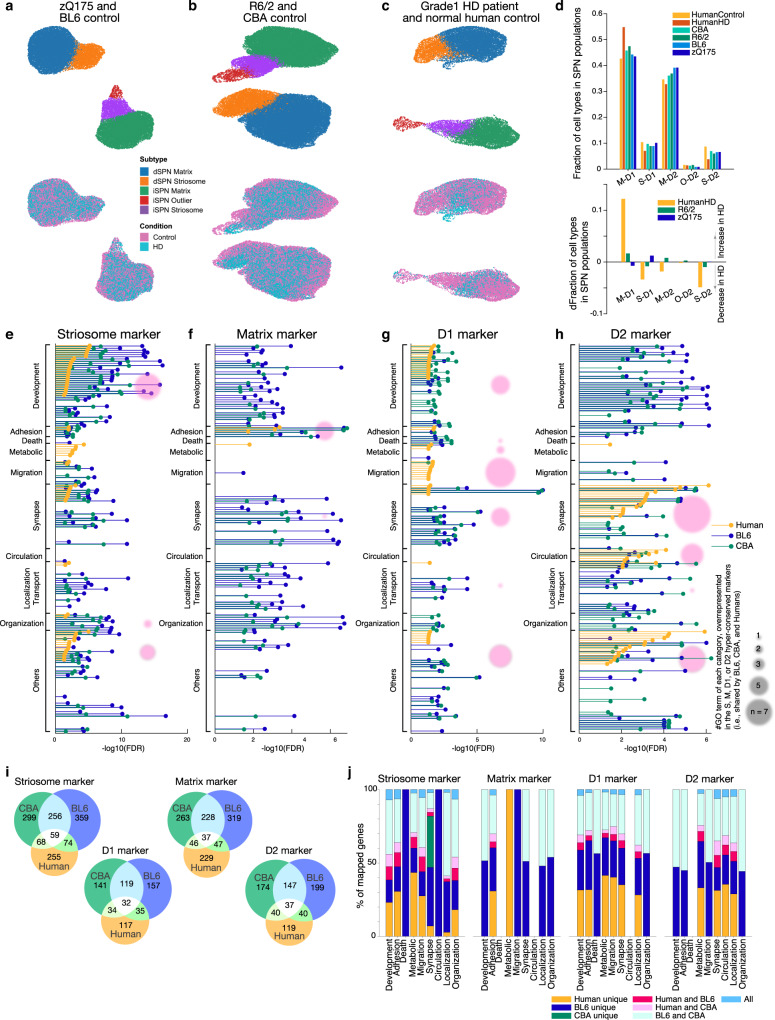
Identification and characterization of human and rodent SPN cell-type-specific markers. **a**–**c** ACTIONet UMAP plots of distinct dSPN (D1) and iSPN (D2) parent clusters, and striosomal and matrix sub-clusters within zQ175 (cyan) and their control (magenta) samples (**a**), R6/2 (cyan) and their control (magenta) samples (**b**), and a human Grade 1 HD patient (cyan) and healthy controls (magenta, **c**). Samples are described in Supplementary Dataset 1. **d** Fraction of each SPN subtype in the entire SPN population identified (top) and the change of fractions in HD as compared to controls (bottom). **e** FDR for enriched GO terms in universal striosome markers found in BL6 (blue) and CBA (green) mice, and in human (orange). Within the GO terms overrepresented in striosome, matrix, D1, or D2 markers, top (i.e., lowest FDR) 40 GO terms are included, and grouped into 9 categories. In each group, the GO terms are sorted by FDRs for humans. Pink circles indicate the number of GO terms in each category, found to be commonly overrepresented in the hyper-conserved striosome markers. **f**–**h** Same as **e** but for universal matrix (**f**), D1 (**g**), or D2 (**h**) markers. **i** Venn diagrams showing the marker overlaps across species and across mouse strains for universal striosome, matrix, D1 or D2 markers as labeled. **j** Overlap of marker genes across species that are mapped onto GO terms related to development, adhesion, metabolic, migration, synapse, circulation, localization/transport, and organization (see Supplementary Dataset 2 for GO IDs included). See also Supplementary Figs. 1–5.

We analyzed snRNA-seq data by using ACTIONet^20–32^ (Supplementary Fig. 1), from striatal samples harvested from human striatum and from zQ175 and R6/2 HD model mice (Fig. 1a–c)^17^. In addition to data originally reported in an initialstudy without attention to the coordinated compartmental transcriptomics examined here, we newly collected and reported here data from a rare Grade 1 case, including from samples of both the caudate nucleus and the putamen. For human snRNA-seq analysis, therefore, we sequenced separately the caudate and putamen samples and combined the new dataset from the Grade 1 case with the previous dataset of control human samples published by Lee et al.^17^, and we re-clustered the combined dataset using striosome-matrix marker genes (Fig. 1c). We collected 62,487 nuclei across twelve controls, and the Grade 1 HD case. For the mouse models, the numbers were 112,295 nuclei across fifteen mice: eight isogenic control and seven R6/2 model mice; and 63,015 nuclei across eight mice: four isogenic control and four zQ175 model mice (Supplementary Dataset 1). R6/2 (and their control, CBA) mice were harvested at 9 weeks of age, and zQ175 (and their control, BL6) mice at 6 months of age. Only male mice were used, given HD model differences in phenotype progression between male and female mice. They were taken at the earliest time point to detect robust transcriptomic and behavioral phenotypes in heterozygous mice based upon previous whole-tissue RNA sequencing studies^4^ and others^33,34^.

With a curated set of markers (Supplementary Dataset 1, Supplementary Fig. 2), we identified striosomal (S) and matrix (M) SPN sub-clusters within both the D1 and D2 clusters in the mice (Fig. 1a, b), as previously described^18,19^, as well as in humans (Fig. 1c). In addition to S and M clusters, we identified, within the D2 cluster, a distinct yet small subcluster, here provisionally named ‘outlier D2’ (O-D2), which appeared in all samples across both phenotypes in both murine and human species. The O-D2 cluster is transcriptomically closer to the striosomal identity than to that of the matrix and shares markers with the ‘D1/D2 hybrid’ recently identified in the non-human primate^31^. On the other hand, O-D2 is likely distinct from ‘eccentric SPNs’^19^. These cell types, identified by ACTIONet^20–32^, co-clustered perfectly atop each other between controls and the HD samples (magenta and cyan dots, Fig. 1a–c), affirming the consistency of cell-type annotations across phenotypes. There were no differences in quality control metrics between them or in the fraction of cells discarded (Methods), indicating that cell identities were not sufficiently perturbed by mutant huntingtin to confound annotation. Correspondence of the cell-type annotations was further supported by the well-matched fraction of each SPN subtype in the entire SPN population across samples (Fig. 1d).

Of note is the profound depletion of SPNs found in the human HD brain, especially, of S-D2 SPNs and S-D1 SPNs, followed by M-D2 SPNs (Fig. 1d). This pattern indicated a clear preferential vulnerability of striosomal SPNs in HD, alongside the well-known D2-predominent vulnerability in this disorder. This is unlikely due to identity switching from striosome to matrix in human HD. The SPNs are simply depleted from the corresponding subclusters identified by ACTIONet (Fig. 1c), which were annotated taking into account thousands of transcripts. We clustered and annotated cell-types by feeding combined datasets including both control and HD samples as shown in Fig. 1c, where the main sub-clusters of S and M within the parent D1 and D2 clusters still could be identified as such. Thus, even if many individual transcripts could have had inverse preferential expression within their overall sub-cluster (corresponding to striosome or matrix), the cell-type identities could be reconstructed according to the other weakly modulated thousands of transcripts. As expected from previous work, in samples from the zQ175 and R6/2 mice, SPN loss was negligible despite the disturbed transcriptional profiles of SPNs.

This S-M compartmental sub-clustering in human snRNA-seq samples prompted us to examine S-M markers for potential conservation across species. We took the ratio of each gene expression level (i.e., fold change, FC) between S-D1 and M-D1, to identify potential striosome markers in the D1 population as genes with differential expressions surpassing abs(log_2_FC) > 0.1 and *p* values (<0.001) adjusted for false discovery rates (FDRs). We identified potential striosome markers in the D2 population with the same criteria in the comparison between S-D2 and M-D2. The overlap of marker genes between human and two rodent lines (BL6 as zQ175 controls, and CBA as R6/2 controls) showed that universal striosome markers, i.e., expressed more highly in striosomes both in D1 and D2 populations, were more conserved than those detected only in either D1 or D2 population (Fig. 1i). Similarly, matrix, D1 and D2 markers were better conserved when they were shared in D1 and D2 or striosome and matrix populations (Supplementary Fig. 3). Overrepresented gene ontology (GO) terms in each of four universal markers were found largely to be mapped into 9 categories of GO terms, related to development, cell adhesion, metabolism, migration, synapse/signaling, blood circulation, localization/transport, and organization (see Supplementary Dataset 2 for GO IDs included). As shown in Fig. 1j, in all 9 groups of GO terms, the identities of mapped genes were partially shared across human and mouse, i.e., identified as markers in humans and one of the rodent strains (magenta and pink) or both strains (dark cyan). Supplementary Fig. 4 shows across-between species correlation of differential expression of S-M markers dependent on compartments (i.e., log_2_FC). The majority of striosome markers in one species were also expressed more highly in striosomes than in matrix in the other species (1st and 3rd quadrants of plots shown in Supplementary Fig. 4), but a noticeable number of markers showed opposite compartmental preference between rodent and human (2nd and 4th quadrants). For example, *CNTN5* is a striosome marker in human, but a matrix marker in rodents. Overall conservation (i.e., overlaps) of gene identities were similar for D1-D2 markers (Supplementary Fig. 4), but D2 markers tended to be more conserved than D1 markers.

We found universal striosome, matrix, D1, and D2 markers to have distinctive patterns of GO enrichment (Fig. 1e–h). Across species, striosome markers (Fig. 1e) overrepresented development-related GO terms, whereas matrix markers (Fig. 1f) overrepresented those related to cell adhesion. In humans, D1 markers (Fig. 1g) overrepresented development- and migration-related GO terms, whereas D2 markers (Fig. 1h) overrepresented those related to synapse/signaling and blood circulation. These cell-type-specific patterns became further obvious when we included only universal marker genes conserved across both strains and mouse-human species for GO analysis (hyper-conserved markers, pink circles in Fig. 1e–h). The hyper-conserved markers include well-known key transcription factors involved in cell-type differentiation or signatures of every cell type after differentiation; *EphaA5*, *Htr2a*, *Oprm1*, and *Rxrg* were hyper-conserved striosome markers, *Epha4*, *Id4*, and *Zfhx3* were those for matrix, *Drd1*, *Ebf1*, *Foxp2*, *Pdyn*, *Reln*, and *Tac1* were those for dSPNs, and *Drd2*, *Oprd1*, and *Penk* were those for iSPNs. These patterns might indicate that, because the generation and maintenance of SPN cell-types are crucial for survival, mutant animals might have been eradicated through evolution if their mutations resided in the key marker genes irreplaceable for the differentiation or manifestation of SPN cell-types.

What does the enrichment of development-related GO terms mean for the identities of striosomes or their function? We further looked into the GO terms overrepresented in the hyper-conserved striosome markers (*n* = 59), matrix markers (*n* = 37), D1 markers (*n* = 32), and D2 markers (*n* = 37). We used ShinyGO 0.76 (http://bioinformatics.sdstate.edu/go/) to identify the enriched GO terms (Supplementary Fig. 5), and confirmed the pattern shown by pink circles in Fig. 1e–h. Development-related GO terms were found to be overrepresented both in hyper-conserved striosome markers and in D1 markers, but there was a difference between them. Striosome markers overrepresented GO terms related to morphogenesis and projection development (*Cdh11, Lgi1, Epha5, Col25a1, Sema6d, Phactr1, Myo3b, Ripor2*), whereas D1 markers overrepresented those related to general development and migration (*Drd1, Erbb4, Reln, Plxna2, Ephb1, Nrg3, Isl1*). Many of these striosome markers were enriched in striosomes compared to matrix more prominently than other striosome markers (log_2_FC > 0.3, Supplementary Fig. 5e), and their dysregulation in HD followed the general rule to obscure striosomal transcriptomic identities; upregulated more in matrix, downregulated more in striosomes (Supplementary Fig. 5f). It is, thus, of interest whether the morphological and projection identities of striosomes are compromised in HD, and responsible for the early symptomatology of HD. Although we could analyze a subset of differentially expressed genes in human, i.e., human striosome markers (Fig. 4) in this study, the small number of surviving SPNs in human HD patients, even in the single Grade 1 case, and the limited availability of their tissues, rendered it impossible to analyze differentially expressed genes using the snRNA-seq data nor the histological/anatomical analysis in depth. Accordingly, we focused our analyses on the zQ175 and R6/2 mouse models.

### Striosome-matrix transcriptomic distinctions are more vulnerable than those of D1-D2 in HD

**Fig. 2:**
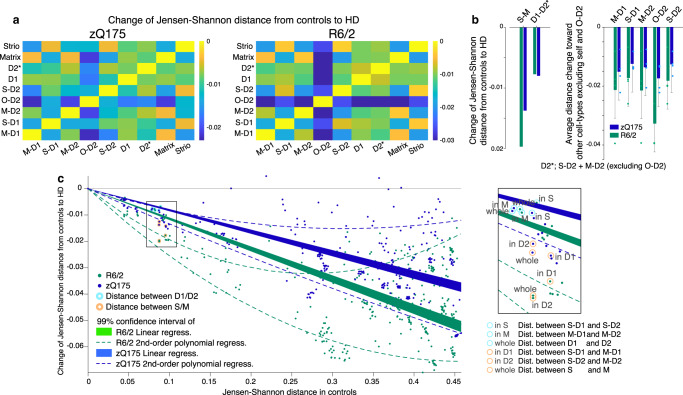
Striosome-matrix transcriptomic distinction is more vulnerable than those of D1-D2 in HD. **a** Jensen-Shannon distances between pairs of cell types for zQ175 (left) and R6/2 (right) as compared to control. Negative values indicate loss of transcriptomic distinction. **b** Summary of loss of transcriptomic distinctions measured by Jensen-Shannon distance. Distinction between striosomes and matrix (S-M) and between dSPN and iSPN (D1-D2*) are shown. Right: Loss of distinction between each pair of cell-types. Blue (zQ175) and green (R6/2) bars indicate average loss of distances from each cell-type (labeled above) toward other cell types (excluding self and O-D2). Thus, the numbers of data points are 4 for all SPN subtypes other than O-D2 (*n* = 3), and all data were plotted as independent measures. D2* does not include O-D2. Error bars indicate 95% confidence intervals. **c** Loss of Jensen-Shannon distances in HD as a function of those in controls are shown for every pair of cell types in the striatum of zQ175 (blue) and R6/2 (green) models. Data points corresponding to S-M distinctions are indicated by orange circles, whereas those for D1-D2 are indicated by cyan circles. Loss of distance (i.e., loss of transcriptomic distinction in HD) was larger for endogenously more distinct cell-type pairs in controls as captured by the 99% confidence intervals of linear regression (shades) or second order polynomial regression (broken lines). Inset: enlarged image of boxed area. Note that loss of distance between S-M populations is larger than expected from the entire striatal dataset, whereas loss of distance between D1-D2 populations is smaller.

In zQ175 and R6/2 models, we compared the expression of each transcript in HD mouse models with that in controls. We found 3,609 genes in R6/2 mice and 2,446 genes in zQ175 mice to be significantly upregulated or downregulated in at least one cell type, as judged by the criteria of abs(log_2_FC) > 0.1, with *p* < 0.001. Figure 2a illustrates HD-associated alteration of Jensen-Shannon distances between each pair of cell types in transcriptional space for the two HD models (see Methods). Distances measured in the zQ175 and R6/2 model mice were subtracted from those in controls. All resulting metrics (Fig. 2a) had negative values, indicating that the difference between transcriptomes for every pair of SPN types (e.g., M-D1 vs. S-D1) was lessened, i.e., they became more alike, in both zQ175 mice and R6/2 mice.

To our surprise, we found that this loss of distinctiveness was greater for the S-M axis of differentiation than for the direct-indirect pathway (D1-D2) SPN axis of differentiation in both zQ175 and R6/2 models (Fig. 2b). The outlier O-D2 cells were not included in D2*, due to the need for their further characterization. To judge whether the loss of distance between compartments was larger than expected from the distance patterns for the entire population of striatal cell-types, not only SPNs, as a control for the possibility that the loss of SPN distance measurements simply reflected such endogenous differences, we plotted the loss of Jensen-Shannon distance in the zQ175 and R6/2 models as a function of endogenous distance between all possible pairs of cell types identified in the control striatum (Fig. 2c). Losses of distance between striosomes and matrix (orange circles) were larger than expected from the linear or second-order polynomial regression of the entire striatal dataset; by contrast, losses of distance between D1 and D2 (cyan circles) were smaller than expected. These results indicate that, of the two classic axes of SPN classification in the striatum, the S-M axis is more imbalanced than the D1-D2 axis in the zQ175 and R6/2 models, and demonstrate that this skewed abnormality coexists alongside the well-known preferential vulnerability of iSPNs in HD.

**Fig. 3:**
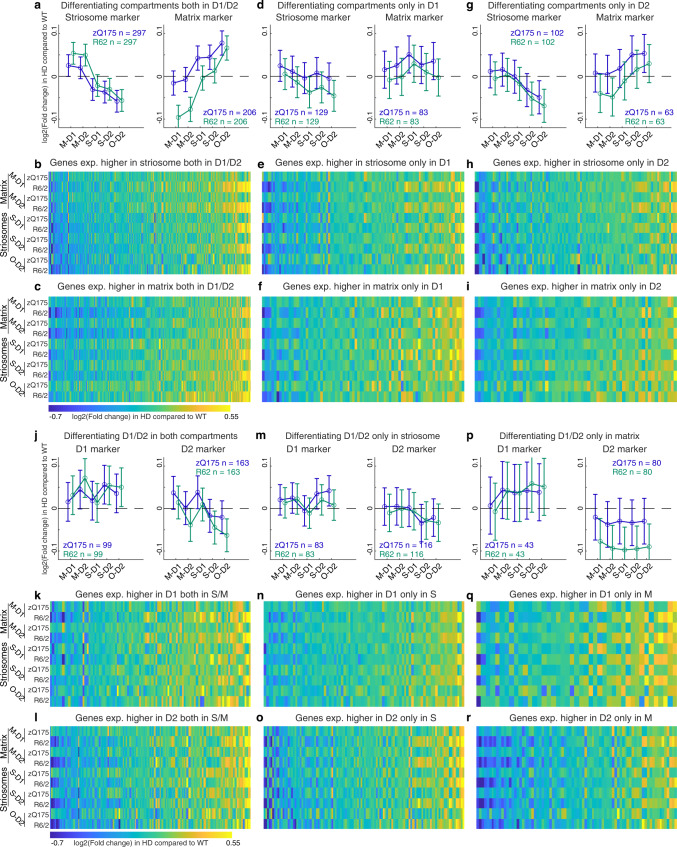
S-M markers but not D1-D2 markers exhibit cell-type-dependent dysregulation to blur their transcriptomic, discriminative identities. **a** Alteration of striosomal (left) and matrix (right) marker expressions, which differentiated the control compartments both in dSPN and iSPN populations in zQ175 (blue) and R6/2 (green) models. N indicates number of markers included in each panel. Error bars indicate 95% confidence intervals for the averages. **b**, **c** Alteration of striosome (**b**) or matrix (**c**) marker expressions in **a**. **d**–**i**, Same as in **a**–**c**, but for markers differentiating the compartments only in D1 (**d**–**f**) or D2 (**g**–**i**) population. **j**, Alteration of dSPN (left) and iSPN (right) marker expression, which differentiated D1-D2 SPNs in both compartments in controls, for each model. N indicates number of markers included in each panel. Error bars indicate 95% confidence intervals for the averages. **k**, **l** Alterations of D1 (**k**) or D2 (**l**) marker expressions in **j**, shown for each cell type of each model. **m**–**r** Same as in **j**–**l**, but for markers differentiating D1-D2 SPNs only in striosomes **m**–**o** or in matrix **p**–**r**. See also Supplementary Fig. 6.

To probe for mechanisms that might lead to blurring of the transcriptomic distinctions along S-M vs. D1-D2 axes, we again focused on the marker genes and examined their upregulation or downregulation (Fig. 3). We identified universal or selective striosome markers as described above, respectively, as those transcripts for which expression was (1) significantly higher in striosomes than in matrix both in dSPN and iSPN populations in controls (S-D1 > M-D1, and S-D2 > M-D2, abs(log_2_FC) > 0.1 and *p* < 0.001), or (2) significantly higher in either of these populations considered singly (S-D1 > M-D1, or S-D2 > M-D2, abs(log_2_FC) > 0.1 and *p* < 0.001). Similarly, we identified universal or selective matrix markers, respectively, as those transcripts for which expression was significantly higher in matrix than in striosomes in both or either in dSPN and iSPN populations in controls (S-D1 < M-D1, and/or S-D2 < M-D2). A consistent pattern emerged: striosome markers were more *up*regulated in matrix SPNs, but were more *down*regulated in striosomal SPNs, for both zQ175 and R6/2 models. Conversely, matrix markers were more *up*regulated in striosomal SPNs, and more *down*regulated in matrix SPNs in both models. The upregulations and downregulations followed a gradient pattern from M-D1, M-D2, S-D1, S-D2, to O-D2 (Fig. 3a–i).

### S-M markers but not D1-D2 markers exhibit cell-type-dependent dysregulation that blurs the transcriptomic, discriminative identities of the striatal SPNs in the HD model mice

These findings indicated that both SPNs in striosomes and SPNs in matrix exhibited a loss in their endogenous identities. This pattern of dysregulation, tending to cancel differential expressions of S-M markers, was clear in those transcripts differentiating S-D2 and M-D2 (Fig. 3a, g) but was not uniformly detectable in those differentiating only S-D1 and M-D1, e.g., both striosome and matrix markers exhibited a tendency to be upregulated in M-D1 of zQ175 mice (Fig. 3d). The patterns held even after applying stricter criteria to define markers; we found similar, even clearer, patterns when we included S-M markers only if they differentiated compartments to the larger degree (abs(log_2_FC) > 0.2 rather than 0.1 and *p* < 0.001, Supplementary Fig. 6). This result indicates that the loss of S-M transcriptomic distinction is not due to the dissipation of weakly differentiating markers, but reflected the core pattern that the prime compartmental markers followed. The abnormality in iSPNs in these zQ175 and R6/2 HD models, thus, included an obscuring of compartmental differentiation between S-D2 and M-D2.

### Loss of compartmental identities is a conserved signature of HD from mice to human

**Fig. 4:**
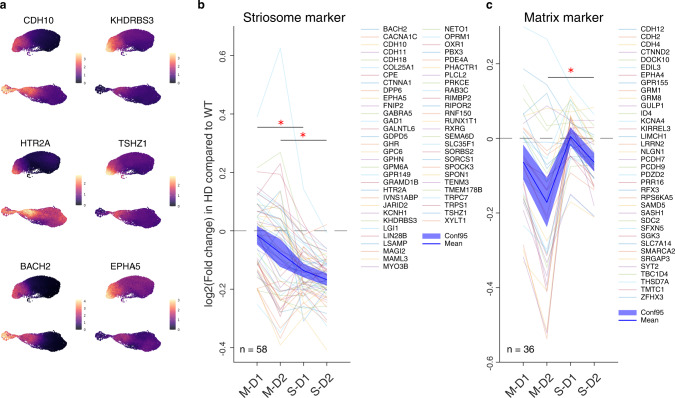
Data from the human Grade 1 HD patient indicate the loss of compartmental identities as a conserved signature of HD. **a** Differential expression of conserved striosome markers is shown for human data including Grade 1 HD patient and controls as in Fig. 1c. Counts of individual transcripts are shown in color codes at right. **b**, **c** Alteration of conserved striosome marker (**b**) and conserved matrix marker (**c**) expressions in the human Grade 1 HD patient relative to controls, shown as log_2_(fold change of the expression in HD as compared to that in controls). N indicates the number of markers included in each panel. One-way ANOVA followed by Tukey-Kramer post-hoc multiple comparison test. One-way ANOVA: *p* = 6.92 × 10^10^, multiple comparison: *p* = 1.14 × 10^6^ for M-D1 vs. S-D1, *p* = 5.120 × 10^4^ for M-D2 vs. S-D2 for conserved striosome marker in **b**. One-way ANOVA: *p* = 2.43 × 10^6^, multiple comparison: *p* = 0.1349 for M-D1 vs. S-D1, *p* = 0.0038 for M-D2 vs. S-D2 for conserved matrix marker in **c**.

Importantly, the loss of transcriptional distinction between striosomes and matrix was also observed in the Grade 1 HD patient. As shown in Fig. 1i, there was a considerable conservation of striosome and matrix markers across species (Fig. 4a). In Fig. 4b, c, we compared the expression level in the Grade 1 HD patient to the control human subjects, for all those conserved compartmental markers (conserved striosome marker: *n* = 58; conserved matrix marker: *n* = 36; one data point was excluded from the analysis for each marker due to insufficient expression levels for adequate analysis). The expression levels of conserved striosome markers were significantly more downregulated in striosomal SPNs as compared to matrix SPNs in both D1 and D2 populations (M-D1 > S-D1 and M-D2 > S-D2, *p* < 0.05, Fig. 4b), whereas those of conserved matrix markers were significantly more downregulated in M-D2 as compared to S-D2 populations, with the consistent trend observed in M-D1 and S-D1 (Fig. 4c), in the Grade 1 patient. These data strengthen our findings in the zQ175 and R6/2 models, suggesting that the transcriptomic distinction between striosomal and matrix SPNs are obscured in HD samples. Thus, the accentuated loss of transcriptomic identities between striosomes and matrix is a conserved signature across human HD patient and murine HD models.

Although the dysregulation pattern to cancel endogenous S-M identities is aligned well across the zQ175 and R6/2 models, one might notice the possible discrepancy in the dysregulation of matrix markers (Fig. 3a, g): matrix markers were noticeably downregulated in M-D1 and M-D2 cells in the R6/2 model, but not in the zQ175 model. In order to delineate the model-specific mechanism overriding the model-invariant pattern, we performed GO analysis of genes that were anomalously upregulated in matrix cells of zQ175 mice, but downregulated in those of R6/2 mice. We found that GO terms related to synaptic transmission and cell-cell adhesion (Fig. 3a) or regulation of immune system processes (Fig. 3g) were overrepresented by the subset of matrix markers that were anomalous by virtue of being upregulated in matrix cells in zQ175 mice. This situation might indicate that synaptic and/or immune modifications are induced specifically by full-length mHTT in the zQ175 matrix at 6 months of age^35^, overriding the model-invariant phenotype to lose transcriptomic matrix identities (Supplementary Dataset 5 for the gene list and GO terms).

In sharp contrast to the compartmental markers, D1-D2 markers did not alter their expression patterns so as to diminish their endogenous identities as dSPNs or iSPNs. Taking genes differentiating D1 and D2 in both or either compartment, D1 markers (S-D1 > S-D2, and/or M-D1 > M-D2, abs(log_2_FC) > 0.1 and FDR-adjusted *p* < 0.001) were inconsistently upregulated or downregulated across S-M and D1-D2 cell types. Thus, upregulation in some genes cancels out downregulation in others, rarely reaching significance in either zQ175 or R6/2 model as a group (left panels in Fig. 3j, m, p). The D2 markers in the matrix of R6/2 mice, but not of zQ175 mice, were significantly downregulated irrespective of D1 or D2 cell types (right panel in Fig. 3p). Thus, in contrast to the clear and robust cell-type-specific dysregulation of S-M markers, dysregulation of D1-D2 marker expressions did not respect cell types, and thus maintained the distinction between D1-D2 populations, even though they distorted the profiles of genes distinguishing these two SPN classes.

### Cell-type-specific dysregulations reflect intrinsic vulnerabilities shared across zQ175 and R6/2 mice

**Fig. 5:**
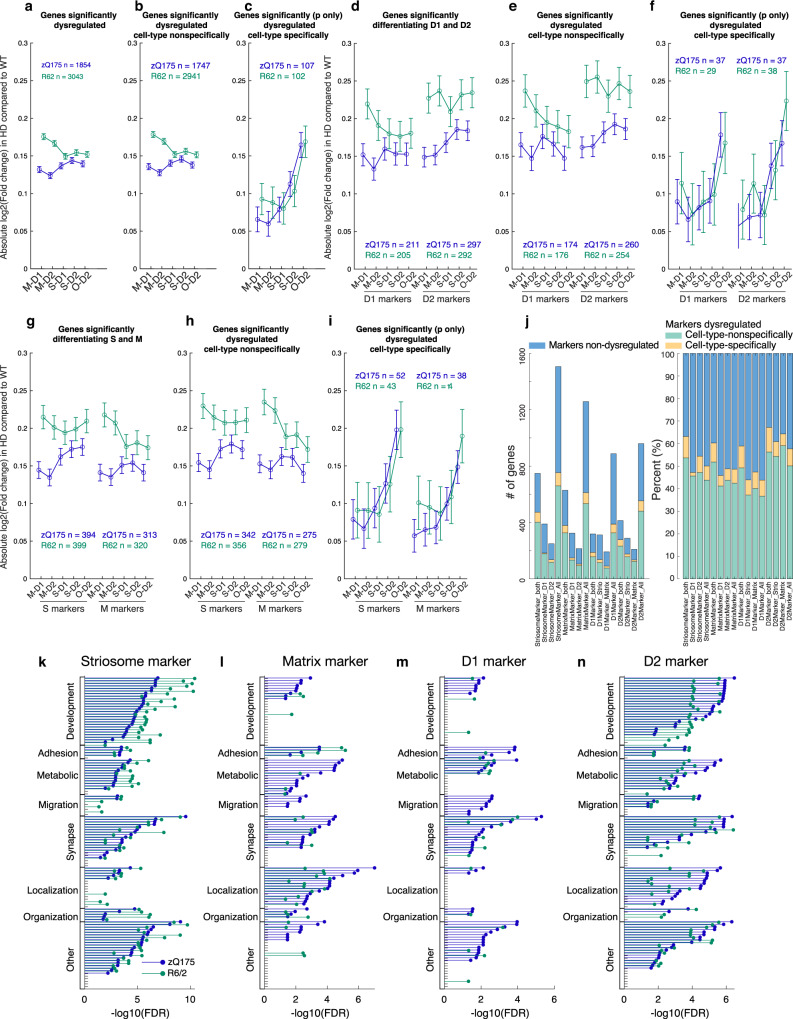
Cell-type-specific dysregulations reflect the intrinsic vulnerability shared across multiple HD models. **a**–**c** Only cell-type-specific gene dysregulations are shared between the two HD models. Average degrees of dysregulation, i.e., absolute value of log_2_(fold change of the expression in HD as compared to that in controls), are shown for all dysregulated genes detected with the criteria of abs(log_2_FC) > 0.1 and *p* < 0.001 in at least one of four canonical cell types (**a**), or the subset of them that are unidirectionally dysregulated (i.e., upregulated, or downregulated in all four cell types, **b**). In **c**, we first selected genes with significant dysregulation (*p* < 0.001) in at least one of four canonical cell types, then further restrict to the genes that are dysregulated bidirectionally dependent on the cell types (i.e., upregulated in one cell type(s) and downregulated in another cell type(s)). N indicates number of markers included in each panel. Error bars indicate 95% confidence intervals. One-way ANOVA followed by Tukey**-**Kramer post-hoc multiple comparison test. **d**–**f**, Same as **a**–**c** but restricted for D1-D2 markers. **g**–**i**, Same as **a**–**c** but restricted for S-M markers. **j**, Composition of patterns of dysregulation for D1-D2 markers and S-M markers. **k**, FDR for enriched GO terms in dysregulated striosome markers in zQ175 (blue) or R6/2 (green) mice. Within the GO terms overrepresented in dysregulated striosome, dysregulated matrix, dysregulated D1, or dysregulated D2 markers, top (i.e., lowest FDR) 40 GO terms are included, and grouped into 9 categories. In each group, the GO terms are sorted by FDRs for zQ175 mice. **l–n**, Same as **k** but showing FDRs of the enrichments in dysregulated matrix (**l**), dysregulated D1 (**m**), or dysregulated D2 (**n**) markers. See also Supplementary Fig. 7.

Next, to identify cell-type-specific alterations, we examined the degree of dysregulation for each cell type. First, we included all marker and non-marker genes if they were dysregulated significantly in at least one cell type in the zQ175 and R6/2 models (i.e., abs(log_2_FC) > 0.1 and *p* < 0.001). Absolute values of log_2_FC in each cell type (Fig. 5a) exhibited more severe patterns of dysregulation in R6/2 than in zQ175, confirming prior observations^17,36^. It is of note, however, that our measurements were made in the context of different ages, i.e., comparing 9-week-old R6/2 mice to 6-month-old zQ175 mice.

Second, we divided the data depending on whether the dysregulation of a given gene was similar across all cell types or differed by cell type. For classification as being in the unidirectional, cell-type-nonspecific category (Fig. 5b), the transcript was upregulated or downregulated in all cell types with the requirement that the dysregulation was significant in at least one cell type (abs(log_2_FC) > 0.1 and *p* < 0.001). For categorization as bidirectional (i.e., cell-type specifically dysregulated; Fig. 5c), the transcript was upregulated in some cell types and downregulated in other types, again with the requirement that the dysregulation be significant (*p* < 0.001) in at least one cell type. The cell-type-nonspecific dysregulations differed depending on which of the zQ175 and R6/2 HD models was examined, whereas cell-type-specific dysregulations were well aligned between the two, reflecting HD-related cell-type-dependent vulnerability held in common. The same rule was consistently observed when we included only D1-D2 marker genes (Fig. 5d–f) or only S-M marker genes (Fig. 5g–i), which were composed of similar proportions of genes dysregulated either cell-type specifically or cell-type nonspecifically (Fig. 5j). The shared pattern of dysregulation across the zQ175 and R6/2 models, measured as degree (i.e., absolute values of differences from their respective controls), had a hierarchy: it was highest in O-D2, followed by S-D2. This hierarchy thus reflected a multiplexing of the D2-dominant vulnerability with compartment-based vulnerability.

In order to gain insight into biological pathways that might be especially dysregulated, we performed a GO analysis applied to (1) marker genes significantly dysregulated in at least one cell type (Fig. 5k–n), (2) cell-type nonspecifically (i.e., upregulated or downregulated in all cell types) or specifically (i.e., upregulated in some cell types and downregulated in other types) dysregulated genes (Supplementary Fig. 7a–c), or (3) marker and non-marker genes dysregulated in each type of SPNs (Supplementary Fig. 7d–h). Consistent with the D2-dominant and striosome-dominant dysregulation, a wide range of GO terms were overrepresented in dysregulated D2 markers (Fig. 5n) and dysregulated striosome markers (Fig. 5k), especially those related to development, which were found to be less overrepresented in dysregulated D1 and matrix markers. The differences in the patterns of enriched GO terms were more alike than those shown for universal S, M, D1, or D2 markers in Fig. 1e–h, indicating that endogenous identities were defined by genes involved in distinct biological pathways, whereas in the zQ175 and R6/2 HD models, dysregulations observed in the marker genes were involved in similar biological pathways. The data analysis thus uncovered nuanced, but considerable, differences in the biological pathways overrepresented in dysregulated S-M and D1-D2 marker genes.

### Histological and physiological loss of compartmental identities in HD model mice

**Fig. 6:**
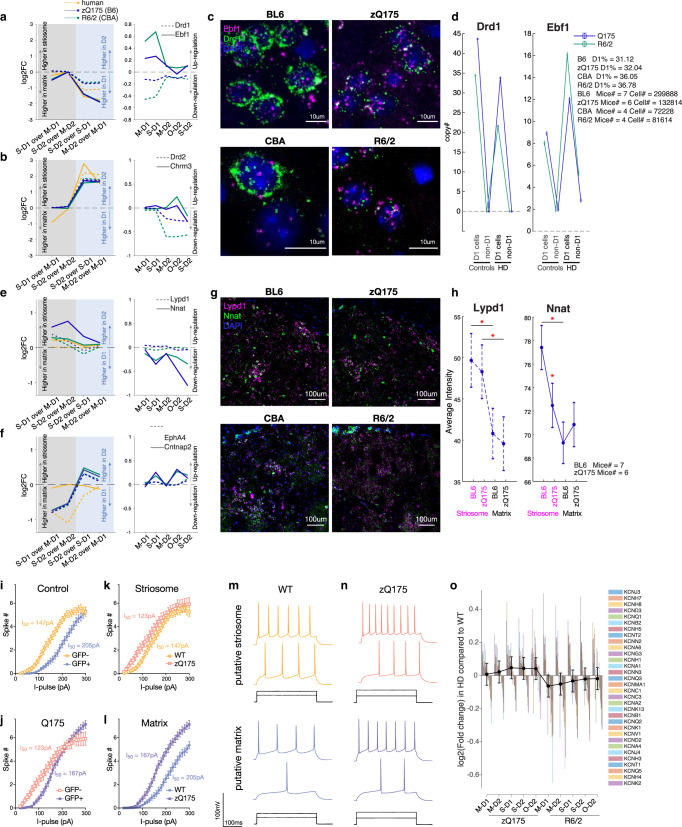
Histological and physiological loss of compartmental identity in HD model mice. **a** snRNA-seq data for dSPN markers, i.e., *Drd1* (broken lines) and *Ebf1* (solid lines). Left: Differential expression was measured between the cell-type pairs indicated below and shown as log_2_(fold change). Right: Expressions in HD models are compared to those in controls. **b** Same as in **a**, but for iSPN markers, i.e., *Drd2* (broken lines) and *Chrm3* (solid lines). **c** FISH images of sections obtained from the anterior striatum in the two HD models compared to their controls, stained for *Drd1* (green), *Ebf1* (magenta), and DAPI (blue). **d** Quantification of FISH image. Copy numbers (i.e., number of detected spots) for *Drd1* (left) and *Ebf1* (right) are shown separately for D1 and non-D1 cells in controls or HD models. Error bars indicate 95% confidence intervals from the averages. **e**, **f** Same as in **a** and **b**, but for striosome markers (**e**), i.e., *Lypd1* (broken lines) and *Nnat* (solid lines), or matrix markers (**f**), i.e., *EphA4* (broken lines) and *Cntnap2* (solid lines). **g** Same as in **c** but for *Lypd1* (magenta), *Nnat* (green), and DAPI (blue). The images were obtained from anterior striatal sections of the same mice shown in **c**. **h** Quantification of FISH image. Average intensity of FISH signals is shown for *Lypd1* (left) and *Nnat* (right) separately for striosomes and matrix in zQ175 mice and in their controls. Error bars indicate 95% confidence intervals. One-way ANOVA followed by Tukey-Kramer post-hoc multiple comparison test. One-way ANOVA: *p* = 0.0001, multiple comparison: striosomes vs. matrix in BL6; *p* = 0.0017, striosomes vs. matrix in zQ175; *p* = 0.0073 for *Lypd1* (left). One-way ANOVA: *p* = 5.89 × 10^7^, multiple comparison: BL6 vs. zQ175 in striosomes, *p* = 0.0052 for *Nnat* (right). **i**–**l** Using zQ175 mice crossed with a matrix reporter mouse line (CalDAG-GEFI-GFP), electrophysiological properties of putative striosomal (GFP-negative, orange) and putative matrix (GFP-positive, purple) SPNs were examined ex vivo. Current-frequency responses are shown for control (**i**) and zQ175 (**j**) mice, or for putative striosomal SPNs (**k**) and putative matrix SPNs (**l**). I_50_ is defined as the input current producing 50% of maximal spike numbers. Error bars indicate SEM. Control: *N* = 10 mice, *n* = 212 neurons. zQ175: *N* = 9 mice, *n* = 198 neurons. The mean ± SD number of cells recorded per each mouse evaluated was 22 ± 6. **m** Representative traces in control mice are shown separately for putative striosomal (top, orange) and putative matrix (bottom, purple) SPNs in response to I_50_ and I_90_ of putative striosomal SPNs. **n** Same as in **m**, but for zQ175 mice. **o** Dysregulation of potassium channels separately shown for each cell-type of R6/2 and zQ175 mice as compared to controls. We included all 32 out of 79 potassium channels whose dysregulations were quantifiable. Error bars indicate 95% confidence intervals from the averages. See also Supplementary Fig. 8.

To verify these transcriptional changes detected by snRNA-seq, we conducted fluorescence in situ hybridization (FISH) for the most prominent dSPNs and striosome markers in the zQ175 and R6/2 model mice (Fig. 6a–h). The sections for FISH analysis were obtained from the same set of mice at matched coordinates (anterior, middle and posterior) in the striatum. *Drd1* was downregulated in dSPNs of HD models (Fig. 6a, c, d), reflecting their loss of transcriptomic identities^37^. However, another strong marker of dSPNs, *Ebf1*, a well-known factor contributing to dSPNs differentiation^38,39^, was upregulated in dSPNs (i.e., M-D1 and S-D1, Fig. 6a), but not in iSPNs, as if to rescue the loss of their identities, which was confirmed by FISH (Fig. 6c, d). *Nnat*, an imprinted gene implicated in brain development^40,41^, is a strong endogenous striosome marker and was downregulated in striosomes of the zQ175 and R6/2 models (i.e., S-D1 and S-D2, Fig. 6e). In FISH data, its expression in striosomes, but not in matrix, was significantly decreased, so as to lose the significant difference between the compartments in zQ175 mice (Fig. 6h). We found, in addition, that *Lypd1*, identified previously as a prototoxin that acts on nAChR as a snake neurotoxin^42^ and as a marker of von Economo neurons^43^, was a robust striosome marker to identify striosomes in zQ175 mice, but it could not do so in R6/2 mice (Fig. 6g), indicating that markers even apparently robust in snRNA-seq data might alter their distribution patterns, hindering identification of compartmental identities by FISH. The low magnification FISH images recapitulate the obscured, blurred transcriptomic distinction between striosomes and matrix in both zQ175 and R6/2 HD models, which could be led by both the downregulation of striosome markers in striosomes and the upregulation of striosome markers in the matrix (Supplementary Fig. 8). It is of note that there were scattered cells with high expressions of *Lypd1* and/or *Nnat* in the R6/2 mice, which might be either displaced striosomal cells, or matrix cells that acquired their expressions.

We also found *Chrm3*, coding muscarinic ACh receptor 3, to be as prominent an iSPN marker as *Drd2* itself (Fig. 6b), and it was upregulated in O-D2 cells in R6/2 mice (but downregulated in S-D2 cells in both zQ175 and R6/2 models). The matrix marker, *Cntnap2*, an autism susceptibility gene^44,45^, with as large a compartmental difference in expression as *Epha4*^46^ (Fig. 6f), was upregulated in SPNs other than matrix SPNs (S-D1, O-D2 and S-D2). Together, these results, confirmed by histological evidence, recapitulate the snRNA-seq pattern that disturbance of compartmental identities was greater than disturbance of D1-D2 identities in the zQ175 and R6/2 HD models (Fig. 6a–h).

These transcriptional changes clearly raised the question of whether they could affect striatal function. As a first attempt to address this question, we conducted electrophysiological experiments in slice preparations aimed at assessing potential differences between striosomal and matrix cells (Fig. 6i–o). We crossed zQ175 mice with CalDAG-GEF1-GFP mice differentially expressing GFP in matrix SPNs^47^. With whole-cell patch-clamp recordings, I_50_, defined as the input current producing 50% of maximal spike numbers, we replicated the higher excitability of putative striosomal (GFP-negative) SPNs as compared to putative matrix (GFP-positive) SPNs in control (Fig. 6i, m; *N* = 10 mice, *n* = 212 neurons, I_50_ = 147pA in GFP-negative SPNs, I_50_ = 205pA in GFP-positive SPNs) as well as in zQ175 (Fig. 6j, n; *N* = 9 mice, *n* = 198 neurons, I_50_ = 123pA in GFP-negative SPNs, I_50_ = 167pA in GFP-positive SPNs) mice. Given that we applied a mixed-effects model equivalent for 2-way ANOVA (see Methods and Supplementary Dataset 6), the difference between GFP-positive and GFP-negative cells are only significant in controls (*p* < 0.0001, mixed-effects model by GraphPad Prism Version 9.3.1), but not in zQ175 (*p* = 0.094), indicating that electrophysiological identities of striosomes and matrix were lost as were their transcriptional identities. When we compared putative striosomal (Fig. 6k) or matrix (Fig. 6l) SPNs across control and zQ175 mice, we found that excitability significantly increased only in putative matrix SPNs (*p* < 0.0001). This elevation of excitability in matrix SPNs might be partly accounted for by the consistent trend across zQ175 and R6/2 HD models for expression of potassium channels to be more downregulated in matrix SPNs than in striosomal SPNs (Fig. 6o, including all 32 out of 79 potassium channels whose dysregulations were quantifiable). Thus, the endogenously less excitable matrix SPNs elevated their excitability significantly to lose the electrophysiological identities represented by the excitability. Here, we see the diminution of functional identities along the S-M axis, as we saw the diminution of transcriptional identities along the S-M axis.

## Discussion

Our findings demonstrate that the two canonical axes of striatal SPN organization, the direct (D1) and indirect (D2) pathway and the striosome-matrix compartmental subdivisions, are inter-dependently and differentially compromised in HD. The findings show that their identities, categorized along the striosome-matrix (S-M) and dopamine receptor D1-D2 axes, are multiplexed, yielding cross-axis vulnerabilities in both the R6/2 and the zQ175 HD mouse models. Our findings further demonstrate, surprisingly, that the transcriptomic distinctions along the striosome-matrix axis were more obscured than those along the D1-D2 axis in both mouse lines. Echoing this pattern, in the Grade 1 HD human striatal snRNA-seq dataset, the S-D2 sub-cluster was the most severely depleted of the entire SPN population, followed by S-D1 and M-D2, and conserved S-M markers were dysregulated to cancel endogenous S-M identities as seen in zQ175 and R6/2 model mice. These consonant findings in the striatum of both zQ175 and R6/2 models and human HD suggest a profound differential susceptibility of the S-M and D1-D2 canonical organizations known to govern the functional and anatomical organization of the striatum. The loss of striosome-matrix identities was found to be conserved across species, from human to mice, and to be intermixed with the known preferential vulnerability of indirect-pathway D2-expressing SPNs.

Our findings are constrained by limitations, some of which we mention here. The mouse samples were harvested from only a single timepoint; 9 weeks of age in R6/2 and 6 months of age in zQ175 models. We therefore cannot disentangle the time course of disease progression or the mechanistic sequence of pathologic events. Further, we have no access to anatomical details of samples that we obtained from the brain banks, except for the tags of the samples as derived from the caudate nucleus or putamen, hindering us from examining subregions of the striatum from which the SPNs were sampled. This is a significant problem, given the well-known regional variations of molecular identities^7,8,18^ and the prototypical pattern of HD progression from the tail of the caudate nucleus forward in the human^48,49^. We also could not fully analyze SPN transcriptomes in the human HD brain samples due to the limited numbers of surviving SPNs even in the Grade 1 HD case and the sample number (*n* = 2, caudate and putamen samples from one patient), which hinders statistical tests to address differential expressions (*n* must be higher than 2). Furthermore, limited tissue availability prevented us from applying FISH or IHC analyses on the human HD case. This difficulty hampered our ability to forecast extension of the results for clinical translation. Nevertheless, the consistence of findings across human HD samples and zQ175 and R6/2 model mice suggests that these limitations did not preclude the appearance of a common pattern of disease vulnerability. A potential link to the clinical findings is that in larger post-mortem samples examined by anatomical methods^13,15^, differential vulnerability of striosomes has been found in HD cohorts from cases of early manifestation and cases of predominant mood disorder symptoms.

We further encountered in our analyses a group of D2-expressing putative iSPNs with an extreme and idiosyncratic transcriptional identity, and we here provisionally refer to them as outlier D2 (O-D2) cells. Their marker genes partially overlap with those of ‘D1/D2 hybrid’ in primate^31^ and ‘eccentric SPNs’ in mice^19^. We treat the identity of O-D2 cells with caution in this study, in as much as they form a continuum not only with classical iSPNs but also with another non-SPN cell-type that expresses *Adarb2*, *Foxp2*, and *Olfm3* (Pineda et al., in preparation). The O-D2 category, although small, was notable in that we found, in the rodent zQ175 and R6/2 models, transcriptomic dysregulations of this class of SPNs to be most severe in the SPN population. However, in human HD samples, O-D2 was not the most affected population; the depletion of O-D2 cells in the Grade 1 HD samples was not as prominent as that of the S-D2 or S-D1 SPNs. Thus, we should be cautious about the mouse-human difference in the vulnerability of O-D2 cells, detected by different metrics, i.e., dysregulation magnitudes of transcripts in mice, and cell loss in humans.

The patterns of cell-type-dependent transcriptional changes that we identified were shared across SPNs in the zQ175 and R6/2 models. Especially in genes dysregulated in opposite directions in different cell types, the cell-type-specific severities of transcriptional alterations were essentially identical in the R6/2 and zQ175 models. This finding supports previous observations^17,50^ indicating that the transcriptomic dysregulations can be in common despite the distinct genetic makeup of these lines; the R6/2 model mice express an N-terminal exon 1 fragment of mHTT, whereas the zQ175 mice express full-length mHTT. As a working hypothesis, we speculate that the cell-type-specific, model-invariant vulnerability stems from the cellular response to the mHTT exon 1‑like fragments, as a direct translation of exon 1 transgene in R6/2 mice, or the products of the proteolysis of full-length mHTT protein translated or from incomplete splicing in zQ175 mice^51,52^. The hierarchical pattern of cell-type-dependent dysregulation, with highest levels in O-D2 followed by S-D2, was exhibited by all genes including S-M and D1-D2 markers and non-markers. The idiosyncratic transcriptomic responses exhibited by the two models were reflected in genes dysregulated in the same direction in all cell types.

It was between the cell-specific markers for the S-M and D1-D2 axes that the patterns of dysregulation were most clearly different in both zQ175 and R6/2 models. Within the S-M axis, the striosomal and matrix neurons each exhibited declines of their own markers but gains of the other’s markers, so that they became less differentiated from one another than in the control mice. Within the D1-D2 axis, by contrast, D1 and D2 markers were altered irrespective of cell type, without diminishing the transcriptomic D1-D2 distinctions, even though the distinctions were distorted. And the obscuring of the S-M axis was more severe in markers found in iSPNs (S-D2 vs. M-D2) than those found only in dSPNs (S-D1 vs. M-D1). These changes could contribute to the preferential abnormality of iSPNs in the zQ175 and R6/2 mice, and at the same time, emphasize the multiplexing of vulnerabilities across the D1-D2 and S-M axes. Potentially important regional distinctions across the striatum remain to be examined.

Our findings open a new view of the disturbance in balance between striosome-matrix and direct-indirect pathway circuits imposed by HD. Our findings open the possibility that these S-M and D1-D2 axes of striatal organization can be subject to distinct pathophysiological alterations. Our data show that the difference in excitabilities, a proxy of electrophysiological identity, across striosomes (higher) and matrix (lower) was diminished by the accentuated elevation of excitability in matrix SPNs in the zQ175 mice. This phenomenon is congruent with the transcriptional analyses, but also could potentially be, at least partly, accounted for by the changes of potassium channel transcripts in zQ175 and R6/2 HD models. Our current data are insufficient for us to comment on whether and when these physiological changes could contribute to behavioral symptoms. Among many possibilities for this intermixed, yet asymmetric vulnerability, we mention one point of interest as it potentially links our findings to the clinical symptomatology of HD from pre-manifest to manifest stages. Our working hypothesis is that via a decrease of normal *HTT*, the S-M disturbance might precede at least in part the D1-D2 disturbance induced by the gain of *mHTT*.

Striosomal SPNs have a characteristic morphology of neurites, confined within and intermingled with each other to define the boundaries of striosomes. This very feature previously allowed us to identify the borders of striosomes under a 2-photon microscope even with the sparseness of fluorescently labeled striosomal cells^53,54^. The arborization of neurites are organized developmentally according to their birthdate from the center (E11-12 born in mice, depending on the striatal district) to the periphery (E13-14 born) of striosomes as their cell bodies do^55^. The birthdate-dependent organization is further found in the striatonigral projection pattern, i.e., E12-13 born SPNs but not E11 or E14 born SPNs project to ventrally extended dopamine dendrites in the substantia nigra pars compacta^55^. Although we have not yet identified how critical these morphological and projectional identities of striosomes are in defining their functions, the genes that are engaged in the cell morphogenesis involved in neuron differentiation, or neuron projection development, are indeed impressively strong striosome markers, well conserved across humans to mice. In zQ175 and R6/2 HD model mice, those striosome markers follow the general rule: upregulated more in matrix, downregulated more in striosomes. Especially, genes that are known to interact with actin networks, such as *Myo3b*, *Phactr1* and *Ctnna1*, are markedly downregulated in striosomes. These results leave some questions, including whether *mHTT* carriers have striosomal cells with different morphology, projections, and thus functions, from early in life, a situation that could account for the early symptomatology in HD. If so, there could be hope for early therapeutic intervention.

Besides the gain of detrimental function of *mHTT*, loss of a beneficial function of wild-type huntingtin has been implicated in HD pathology^53,54,56^ and is gaining interest in response to early clinical failures of antisense candidates^57^. We do not here have direct data that allow us to distinguish effects of lowered levels of normal huntingtin from that of gain of *mHTT* in our samples, but our results are consonant with previous studies. Although full deletion of *Htt* is lethal, mice can live if the expression of a single allele of *Htt* is rescued at P21; they exhibit deficient compartmental organization and develop heterotopias expressing both matrix and striosome markers^58^. This abnormality in striosome-matrix compartmentalization was reported in the context of a homozygous, normal allele deletion during development; we here report compartmental dysregulation, even in the context of heterozygosity, which should be milder but is akin to the condition of HD individuals, who are heterozygous for *mHTT*. Thus, loss of function could also, concomitant with the gain of *mHTT* function^54,59^, hinder the differentiation of striosomal and matrix SPNs. Such evidence is unavailable for human, but studies using induced pluripotent stem cells derived from HD patients^60^ have demonstrated delayed differentiation and an increased pool of striatal progenitors. Abnormality has further been detected by MRI in non-juvenile CAG expansion carriers as young as 6 years of age^61^.

By influencing development, the loss of a normal *HTT* allele in HD heterozygous individuals could hinder the anatomical compartmentalization of the striatum as well as the differentiation of SPNs to acquire transcriptional identities as striosomes (mostly earlier born) or matrix (mostly later born). By contrast, preferential disturbance of D2-expressing iSPNs, known to be positively correlated with motor manifestation and vast transcriptomic alteration in iSPNs, is detected later, just around the age of manifest onset, under the control of CAG repeat length^2,4,5^. Such intermixed but temporally staged disturbance of the S-M and D1-D2 axes would align with mood and cognitive disorders in the pre-manifest stage, and motor disorder in clinically manifest stage, of HD. If so, the decline of transcriptomic differences between striosomes and matrix might exist far earlier than the manifestation of clinical symptoms, even before birth. If the dysregulation of D1-D2 markers reflect the failure of compensatory mechanisms, or the response to somatically expanded CAG repeats^62^, then the D1-D2 dysregulations observed here might not be detectable early in life. These are among critical issues raised by our findings that need resolution in advancing HD therapeutic strategies.

How these data could help in understanding the temporal events or treatment of HD is difficult to evaluate without access to human brain material from early ages. But further steps could be taken in non-human primate models currently being developed, and from zQ175 and R6/2 HD model mice, potentially giving insight to treatment protocols or preclinical testing relevant to non-motor as well as motor biomarkers and symptoms. It would be of great value to identify candidate genes to help prevent S-M differentiation deficits, and to correlate the transcriptomic changes with early-stage non-motor clinical observations. Assessing cell-type-specific gain of function phenomena still faces obstacles difficult to overcome, for example, to measure somatic expansion of CAG repeats within individual cells. In our dataset, the well-known genes that are related to somatic expansion, i.e., *MLH1*, *MLH3*, *FEN1*, *MSH3*, and *MSH6*, do not or only weakly differentiate S-M compartments or D1-D2 cells. Thus, the expression levels of DNA repair pathway genes in single nuclei at adulthood should not be assumed to account fully for the differential susceptibility to somatic expansion or the vulnerability to mHTT aggregates. Technological advances and expansion of databases will not only advance symptom-directed therapeutic strategies for HD, but also other non-motor neuropsychiatric diseases that could harbor compartmental differentiation deficiency. Currently there are limitations in how to translate these findings to a clinical level. New methods, more cases, and deep study of conserved properties of the D1-D2 and S-M axes could well point to developmental vulnerabilities that could inform the clinic.

## Methods

### Animals

All mouse husbandry and experimental procedures were conducted with the approval of the Massachusetts Institute of Technology Committee on Animal Care (CAC protocol #: 1120-114-23 and 1220-115-23). Mice were housed under pathogen-free conditions, with food and water provided ad libitum on a standard 12-h light/12-h dark cycle. Room temperature was maintained at 21 ± 2 °C, and the humidity between 30 and 70%. No procedures were performed on the mice prior to the outlined experiments. For all studies, littermate mice were group-housed, and male littermates were used at ages described in the Method Details and figure legends. Only male mice were used given HD model differences in phenotype progression between male and female mice. Mice were assigned to experimental groups based on their genotype (all mice were used), and as individual biological replicates. B6CBA-Tg(HDexon1)62Gpb/1 J mice (CAG repeat length 160 ± 5; Jackson Laboratories stock # 002810) were used as R6/2, and B6J.zQ175DN (Jackson Laboratories stock # 370832) knock-in congenic C57BL/6J mice were used as zQ175 model. Replicate number per mouse group and sample size was as reported previously^17^.

### Human samples

Post-mortem tissue samples from the caudate nucleus and putamen of Grade 1 HD and from matched, unaffected controls were obtained from the NIH NeuroBioBank or the University of Alabama at Birmingham. The policies and procedures of each brain bank have been reviewed and approved by their respective Institutional Review Boards (IRBs) and any additional appropriate oversight committees of the Brain and Tissue Repositories site’s home institution. Trained individuals request and document consent for brain tissue donation from the deceased’s next-of-kin or legally authorized representative. Individual requests for release of medical records, questionnaires, and/or interviews with individuals knowledgeable of the deceased are obtained according to IRB-approved policies and procedures. A complete inventory of IRBs that oversee the bank and tissue repositories is available at NIH NeuroBioBank [https://neurobiobank.nih.gov]. No non-HD pathologies were reported among HD cases. Controls were considered pathologically normal by the brain bank. There was not a co-occurring neurological disease reported. Human tissue analyses were conducted as exempt human research, as this was secondary research using bio-specimens not specifically collected for this study. All samples were obtained from biobanks/repositories using appropriate de-identification and under consent.

### snRNA-seq and analysis

Nuclear isolation was performed as described in Lee et al.^17^ (*n* = 62,487 nuclei across twelve unaffected control and Grade 1 HD caudate and putamen samples; *n* = 112,295 nuclei across fifteen mice: eight isogenic control and seven R6/2 model mice, all at 9 weeks of age; *n* = 63,015 nuclei across eight mice: four isogenic control and four zQ175DN model mice, all at 6 months of age; samples described in Supplementary Dataset 1). Droplet-based snRNA sequencing libraries were prepared using the Chromium Single Cell 3′ Reagent Kit v3 (10× Genomics, Pleasanton CA) according to the manufacturer’s protocol and sequenced on an Illumina NextSeq 500 at the MIT BioMicro Center (zQ175DN mouse samples) or a NovaSeq 6000 at the Broad Institute Genomics Platform (R6/2 mouse samples and human samples). FASTQ files were aligned to the pre-mRNA annotated Mus musculus reference genome version GRCm38 or human reference genome GRCh38. Cell Ranger v6.0 (10× Genomics, Pleasanton CA) was used for genome alignment and feature-barcode matrix generation.

We used the ACTIONet and scran R packages to normalize, batch correct, and cluster single-nucleus gene counts. Batch-corrected data were used as input to the archetypal analysis for cell type identification (ACTION) algorithm^20–32^ to identify a set of landmark cells or ‘archetypes’, each representing a potential underlying cell state. Using ACTION-decompositions with varying numbers of archetypes, we employed the ACTION-based network (ACTIONet) framework^20–32^ to create a multi-resolution nearest neighbor graph. A modified version of the stochastic gradient descent-based layout method was used in the uniform manifold approximation and projection (UMAP) algorithm^63^, to visualize the ACTIONet graph. A curated set of known cell-type-specific markers (Supplementary Dataset 1) was used to annotate individual cells with their expected cell type and assign a confidence score to each annotation, and network connectivity was used to correct low-confidence annotations. Multiple iterations of this process were performed to identify and prune low quality cells. At each iteration, we removed cells with high mitochondrial RNA content (>5% for mouse and >20% for human), abnormally low or high RNA content (relative to the distribution of its specific cluster with an initial global cutoff of 500 unique genes), ambiguous overlapping profiles resembling dissimilar cell types (generally corresponding to doublet nuclei), and cells corresponding to graphical nodes with a low k-core or low centrality in the network (generally corresponding to high ambient RNA content or doublet nuclei).

In the higher grade HD data reported in Lee et al.^17^, the depletion of SPNs spans across all SPN subtypes, thus making it difficult to estimate with accuracy the fractions of individual subtypes that can be compared to controls. Even in control data, due to the unknown location of brain samples obtained from the brain bank, the variance is large, which also hindered meaningful interpretation of the results. Thus, we did not include the corresponding data shown in Fig. 1d for higher grade HD patients.

Cell-type-specific pseudobulk differential gene expression (DGE) analysis was performed using ACTIONet and limma^64^ for sufficiently abundant cell types using age (human), sex (human), and disease (human and mouse) phenotype as design covariates and gene-wise single-cell-level variance as weights for the linear model. Genes were considered differentially expressed if they had an FDR-corrected *p* value < 0.001 and an absolute log_2_-fold change >0.1 for that cell type relative to the normal control group or the reference cellular sub-type. To ensure that DGE results were reproducible and robust to differences in cell type abundance, we sampled with replacement equal numbers of mice/individuals and cells per mouse/individual for each cell type and repeated the pseudobulk analysis. Lastly, we repeated the analyses using DESeq2 as the model-fitting algorithm in lieu of limma to ensure replicability across methods. In all cases, DGE results were consistent, and we used the pseudobulk limma results for all downstream analyses.

To determine transcriptomic distance, we computed the average gene expression vector for each subtype and calculated the pair-wise Jensen-Shannon divergence (JSD) using the philentropy R package between all subtypes. The JSD is a measure of similarity between two distributions in the interval [0, 1] with 0 denoting two identical distributions. The Jensen-Shannon distance was defined as the square root of the JSD. For Fig. 2, we used the difference of this distance between phenotypes relative to the control to determine the extent of transcriptional identity loss in HD, with more negative values suggesting greater loss of identity.

### GO analysis

All GO analysis used the PANTHER overrepresentation test (Released 20210224)^65^ and Gene Ontology database DOI:10.5281/zenodo.5228828 Released 2021-08-18, FISHER test (http://geneontology.org/). We searched for overrepresented ‘biological pathway’ GOs, corrected with false discovery rate. We used custom reference lists which only contain genes that were detected at all in the experiment in a given species in a given comparison analysis, to correct any possible bias originating from the experimental and/or analytic procedures.

As shown in Figs. 1 and 5, GO terms found to be overrepresented were categorized into 9 groups: development, cell adhesion, metabolism, migration, synapse/signaling, blood circulation, localization/transport, and organization. We categorized GO terms based on the keywords that they include, namely, development, genesis, differentiation, growth or generation for development category, synapse, synaptic, membrane potential, action potential, signaling or signal transduction for synapse/signaling category, adhesion for cell-adhesion category, metabolic, catabolic, biosynthetic for metabolism category, taxis, locomotion, motility, migration for migration category, blood, vasodilation, circulation, or circulatory for blood-circulation category, localization or transport for localization/transport category, and organization or assembly for organization category. Exact GO terms assigned to each category is listed in the TermMembers sheet in Supplementary Dataset 2.

### Histological analysis

Mice were deeply anesthetized and then transcardially perfused with phosphate-buffered saline (PBS) followed by 4% paraformaldehyde in PBS. Brains were post-fixed in the same fixative for 24 h and cryoprotected in 30% sucrose for 48 h at 4 °C, and sectioned 10 µm thick for FISH using a freezing microtome. For FISH, we used RNAscope® Fluorescent Multiplex Detection Reagent (ACD Bio, 320851) to detect Nnat (432631), Lypd1 (318361-C3), Drd1 (461901-C2), Ebf1 (433411), and Chrm3 (437701-C3) following the manufacturer’s protocol.

### Slice preparation

Mice were euthanized by decapitation under isoflurane anesthesia. The brain was rapidly removed and cooled in ice-cold oxygenated cutting N-methyl-D-glucamine (NMDG) solution composed of (in mM): NMDG 105, HEPES 20, KCl 2.5, glucose 5, CaCl_2_ 0.5, MgSO_4_ 10, NaH_2_PO_4_ 1.2, NaHCO_3_ 26, sodium pyruvate 3, sodium ascorbate 5, thiourea 2, buffered to pH 7.4 with HCl. Parahorizontal slices (300 µm) were prepared using a vibratome (Leica VT1000S; Leica Microsystems) and allowed to recover at 32 °C in oxygenated cutting NMDG solution for 10 min. After this recovery period, slices were transferred to a holding chamber containing normal ACSF composed of (in mM): NaCl 124, KCl 3.5, NaH_2_PO_4_ 1.2, NaHCO_3_ 26, Glucose 11, MgSO_4_ 1.3, CaCl_2_ 2.5, (pH 7.3-7.4, osmolarity ~300 mOsm). Slices were allowed to recover for 1 h at room temperature post-slicing before recording commenced. When we performed whole-cell patch-clamp experiments accompanied by membrane break-through and intracellular dialysis, access resistance was measured every 1-2 min to ensure the quality of measurements. Only cells with initial Ra of <30 MOhm, which deviated < ±20% over the period of recording, were accepted. We observed a skewed distribution of membrane resistance measured from the same population of cells, and as they met the quality control measures described above, we included data from all cells.

Recordings were made in total from 212 SPNs in 10 control mice and from 198 SPNs in 9 heterozygous mice. The mean ± SD number of cells recorded per each mouse evaluated was 22 ± 6. Approximately equal numbers of GFP-positive and GFP-negative SPNs were recorded per mouse. Data summarized in Fig. 6i–l were taken from distinct cells.

For the analysis shown in Fig. 6i–n, firing frequency curves were derived from spike number evoked during 10 pA depolarizing steps (300 msec). The application of depolarizing current was manually terminated when evoked spike number reached a stable maximal level in a given cell, so as to prevent channel inactivation. Thus, there are missing values, which prevents pairing to perform repeated measures ANOVA. As implemented in and recommended by GraphPad Prism Version 9.3.1, we analyzed the data by mixed-effects model. This mixed-effects model uses a compound symmetry covariance matrix and is fit using Restricted Maximum Likelihood (REML). In the absence of missing values, this method gives the same *p* values and multiple comparisons tests as repeated measures ANOVA. In the presence of missing values (missing at random), the results can be interpreted like repeated measures ANOVA. We also asked Prism to use the Geisser-Greenhouse correction. We, first, applied the mixed-effects model to all data including both cell-types and both genotypes across all ranges of input current, followed by Tukey’s multiple comparison without assuming sphericity. Geisser-Greenhouse’s epsilon was 0.8097 for this test. We next applied the mixed-effects model, assuming sphericity, to data from control and heterozygous mice, separately, or GFP-positive and GFP-negative cells, separately, followed by Sidak’s multiple comparison.

### Transcriptional profiling

Use of the relevant pipelines for quantification, determination of normality of data, and appropriate statistical analysis of snRNA-seq data are as described previously^17^. Data met assumptions of the statistical approach based upon the experimental design in each case. Differential gene expression analysis of the snRNA-seq data was performed on a by-cell-type basis using both the Wilcoxon rank-sum test and Welch’s t test, using both limma and DESeq2, in order to independently confirm statistical results.

As defined in the main text, expression differences were considered significant if they had an abs(log_2_FC) > 0.1, with FDR-adjusted *p* < 0.001.

### Data analysis of FISH data

We used HALO (Indica labs, v3.3.2541.262) to analyze data taken by TissueFAXS Whole Slide Scanning System from TissueGnostics (Zeiss 20 × 0.5 NA EC Plan-NEOFLUAR objective, Hamamatsu Orca Flash 4.0 V2 cooled digital CMOS camera C11440-22CU for fluorescence imaging, Lumencor Spectra X light engine, motorized stage). To prepare for semi-automated image analysis, we exported all images taken by TissueFAXS systems in 16-bit format with the full range of bit color intensity values (0–65536).

For the analysis shown in Fig. 6c, d, we applied customized algorithm modified from Indica_Labs_-FISHIF v2.1.5 to data from dorsal striatal regions (anterior, mid, and posterior sections from each animal). Briefly, we set a threshold (including contrast threshold, intensity, segmentation aggressiveness, size and roundness) to detect nuclei by using DAPI channel, and marked surrounding cytoplasm. For *Drd1* and *Ebf1*, we individually optimized contrast threshold, intensity, spot size, and segmentation aggressiveness to detect and count each copy of mRNA signals. We then extracted copy numbers of *Drd1* and *Ebf1* in individual cells identified from DAPI signals and defined dSPNs as cells with *Drd1* copy # >4. We tried different thresholds of *Drd1* copy # to define dSPNs (e.g., 2, 6, 10), to confirm that the results were largely the same.

For the analysis shown in Fig. 6g, h, we applied customized algorithm modified from Indica_Labs_-Area Quantification FL v2.1. Briefly, we manually scored the *Lypd1* channel of each section (dorsal striatal regions at anterior, mid, and posterior levels from each animal), to annotate striosome areas (i.e., regions of interest, or ROIs) as *Lypd1*-positive areas. To define matrix ROIs, we copied the size and shape of the nearby corresponding striosome ROI, and pasted the ROI to the surrounding *Lypd1*-negative region. We then measured the average intensities of *Nnat* and *Lypd1* in the striosome and matrix ROIs.

### Data analysis of ex vivo experiments

All data were analyzed with Clampfit (Molecular Devices) and/or customized MATLAB routines. Both genotypic and GFP-positive/negative comparisons were made using a non-parametric ANOVA (Kruskal-Wallis) followed by a Dunn’s post-hoc test. Data for firing frequency curves are presented with mean ± SEM. Firing frequency curves were analyzed using GraphPad Prism Version 9.3.1.

### Statistics & reproducibility

Sample size for mouse studies was determined based on sufficient statistical power obtained in the similar prior studies^17,66^. The size of human samples was restrained by the sample availability of the rare and precious Grade 1 HD post-mortem brains, but whenever possible, set to be >3. No statistical method was used to predetermine sample size. All data that met the criteria for quality control were included in the analyses.

### Reporting summary

Further information on research design is available in the Nature Portfolio Reporting Summary linked to this article.

## Supplementary information

Supplementary Information

Description of Additional Supplementary Files

Supplementary Data 1

Supplementary Data 2

Supplementary Data 3

Supplementary Data 4

Supplementary Data 5

Supplementary Data 6

Reporting Summary

## Data Availability

All sequencing datasets used in this study were generated in Lee et al.^17^, except for human Grade 1 data. We generated the human Grade 1 data in this study and have added them to the accession # GEO: GSE152058, which contains the data previously generated in Lee et al.^17^. We re-analyzed these data in this study with the curated set of compartmental markers, which allowed us to identify striosomal and matrix subclusters and thus to extend and deepen the analysis reported in the previous study. The processed differential expression gene data are available at 10.6084/m9.figshare.21677510.v2.

## Code availability

Codes used to construct ACTIONet and identify cell types are accessible from https://github.com/shmohammadi86/ACTIONet.
