# Supplementary material for: Transcriptional vulnerabilities of striatal neurons in human and rodent models of Huntington’s disease
Source: Nat Commun. 2023 Jan 17;14:282. doi: 10.1038/s41467-022-35752-x (PMC9845362; doi:10.1038/s41467-022-35752-x)
Supplement: Supplementary file 1 — Supplementary Information [file 41467_2022_35752_MOESM1_ESM.pdf]

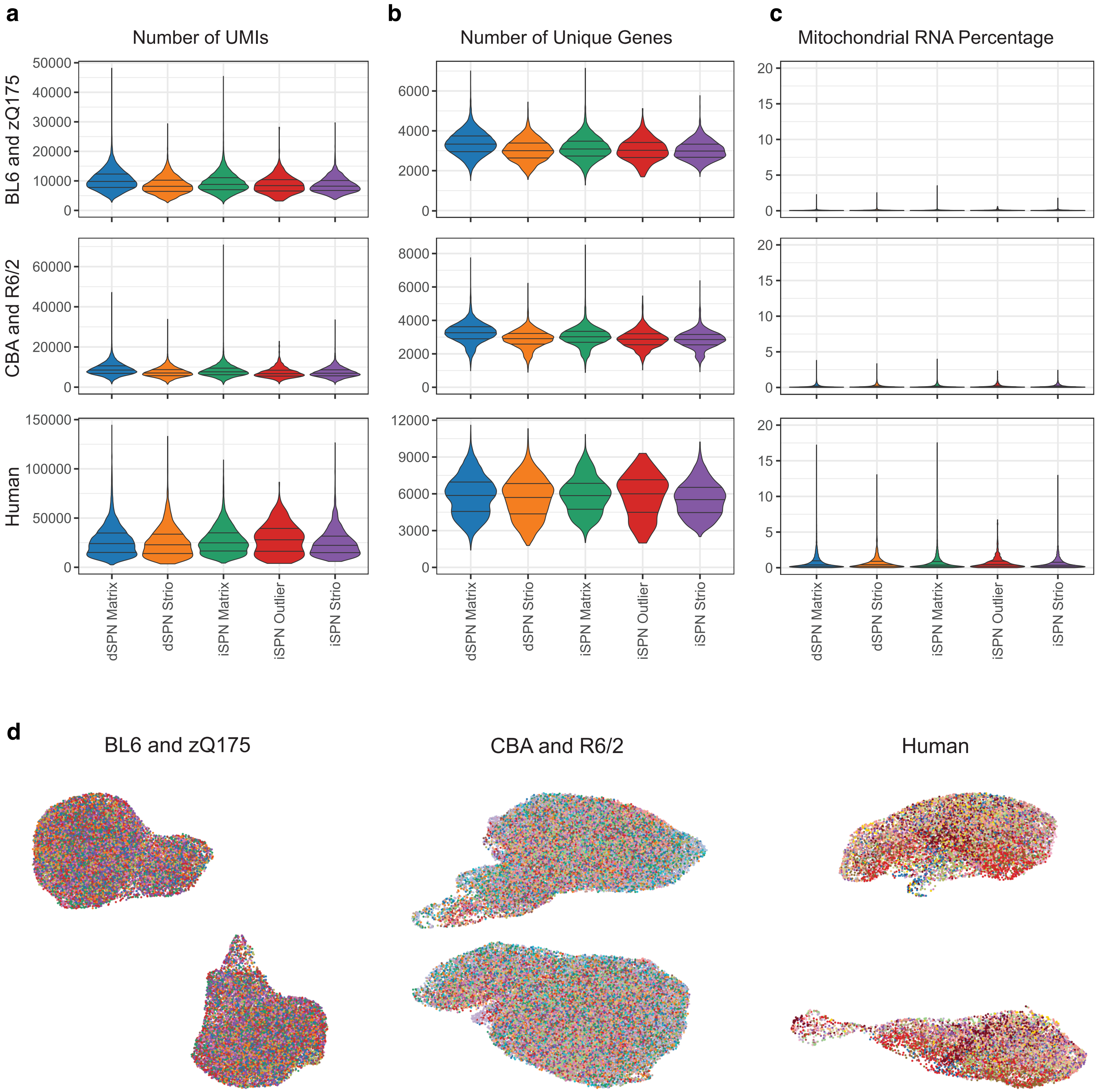

Supplementary Fig. 1 | Benchmarking of snRNA-seq and data analysis by ACTIONet in this study. a-c, Quality control metrics of snRNA-seq data, with the number of unique molecular identifiers (UMIs; i.e., unique transcripts, **a**), number of unique genes (**b**), and percentage of mitochondrial RNA (**c**) per cell type. **d,** ACTIO-Net UMAP plots shown in Fig. 1 colored by individual samples of caudate nucleus and putamen.

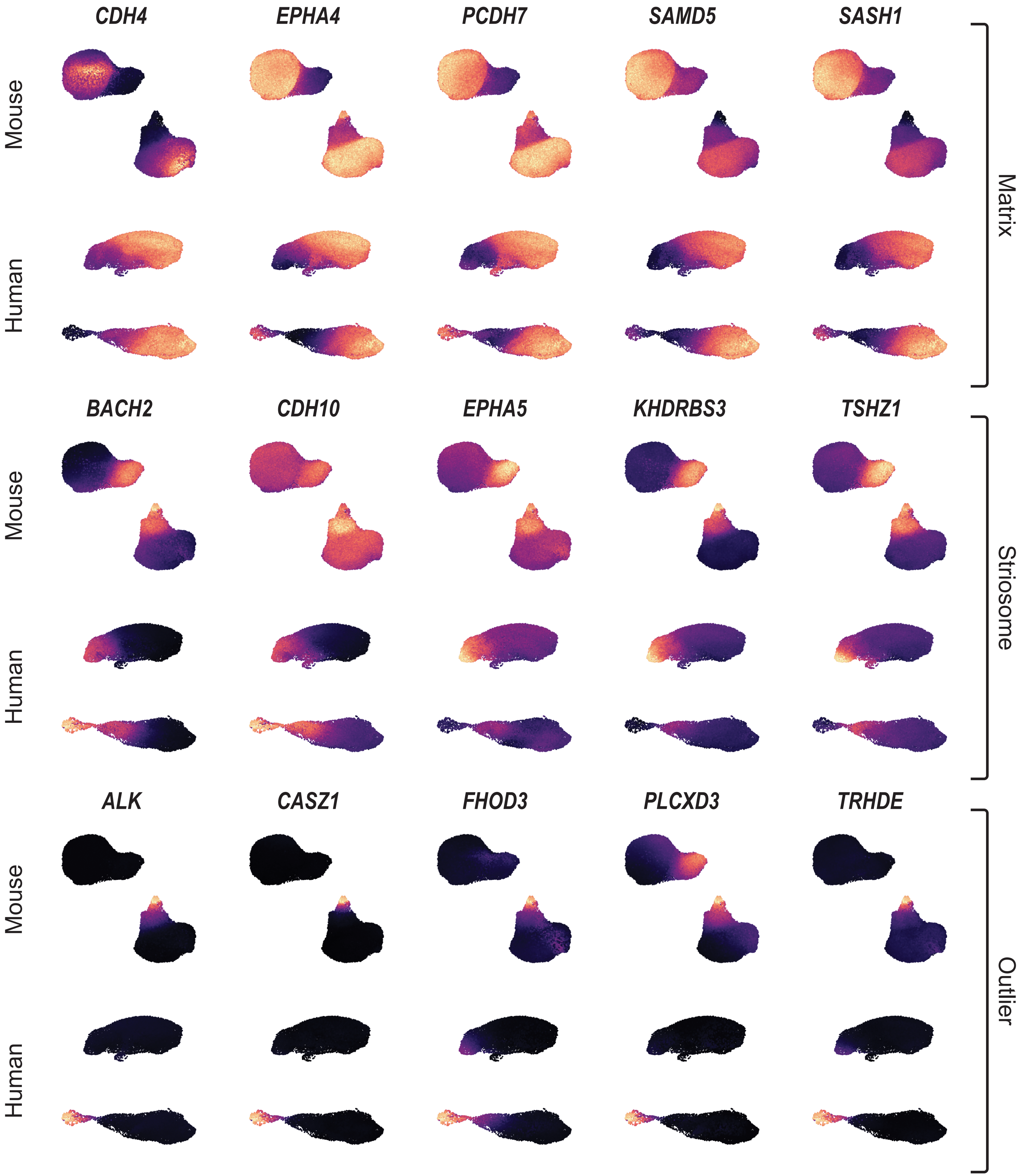

Supplementary Fig. 2 | Expression of cell-type specific markers. Relative expression of representative matrix, striosome, and iSPN outlier marker genes in human and mouse (BL6 and zQ175) snRNA-seq datasets shown in Fig. 1.

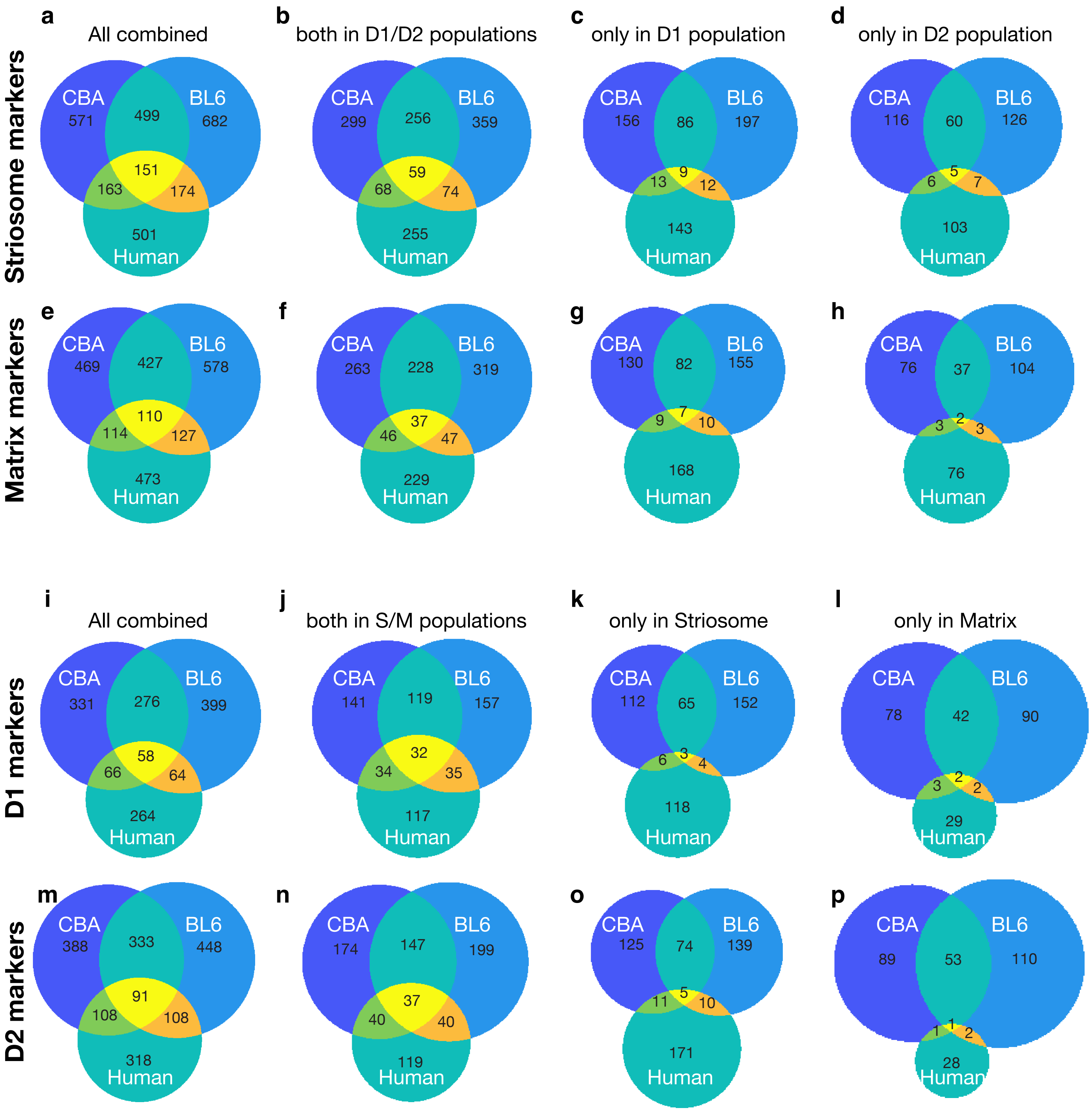

Supplementary Fig. 3 | Genes differentiating S/M in both D1/D2 populations are more conserved than those differentiating S/M only in either population. a-d, Venn charts showing the shared and unique striosome markers (i.e., expressed higher in striosomes than in matrix), detected in D1 and D2 population (**b**), only in D1 population (**c**), only in D2 population (**d**), or listed as either one of the three (**a**). **e-h,** Same as **a-d**, for matrix markers (i.e., genes expressed higher in matrix than in striosomes). **i-l,** Same as **a-d**, for D1 marker (i.e., genes expressed higher in D1 than in D2 cells). **m-p,** Same as **a-d**, for D2 marker (i.e., genes expressed higher in D2 than in D1 cells).

Note that universal markers, i.e., S/M markers that are consistently detected in D1 and D2 population (**b, f**) or D1/D2 markers that are consistently detected in both compartments (**j, n**), are more conserved than markers differentiating only in a specific SPN population.

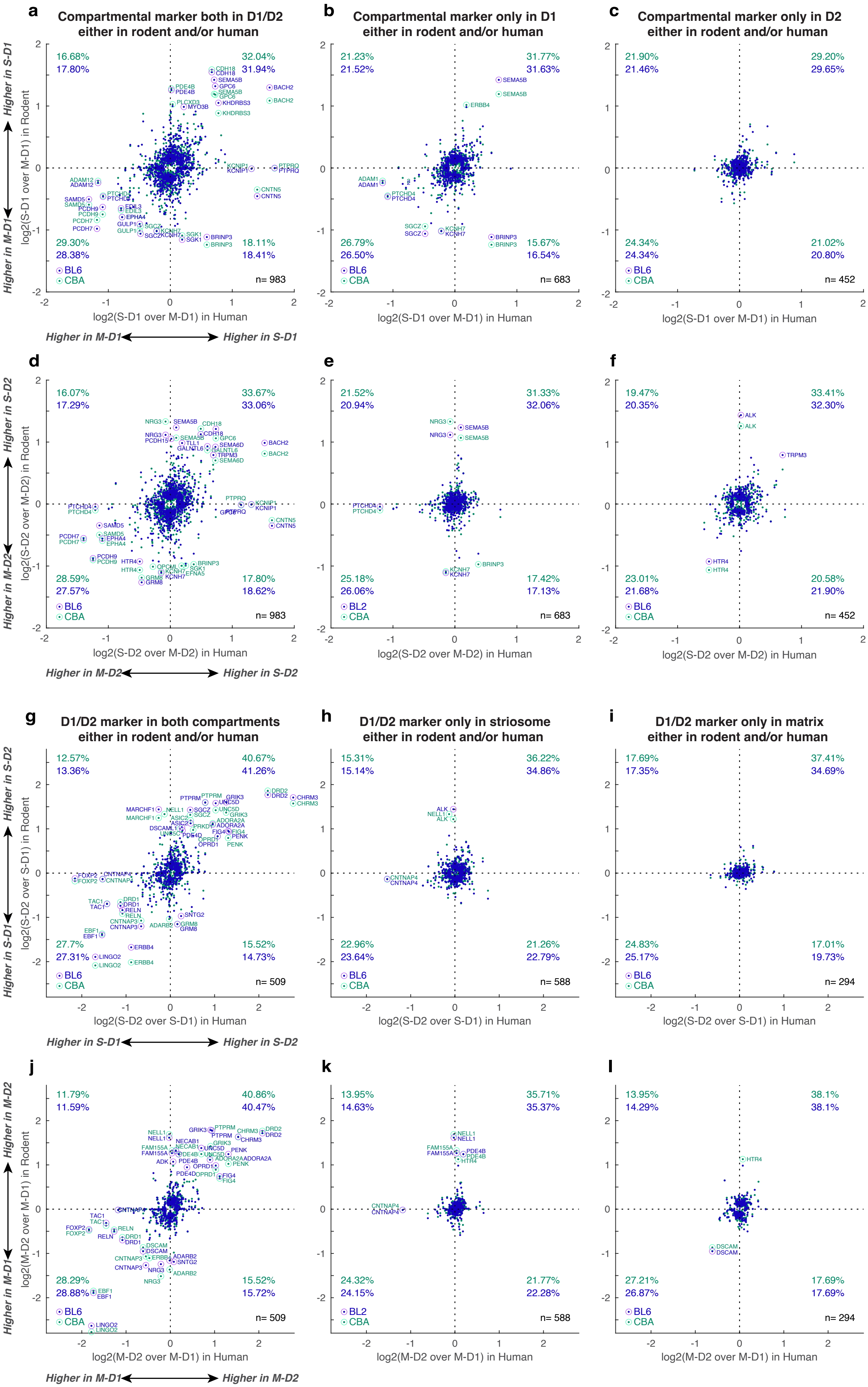
a
Compartmental marker both in D1/D2 either in rodent and/or human
16.68%
17.80%
32.04%
31.94%
29.30%
28.38%
18.11%
18.41%
BL6
CBA
n= 983
log2(S-D1 over M-D1) in Rodent
log2(S-D1 over M-D1) in Human
Higher in S-D1
Higher in M-D1
b
Compartmental marker only in D1 either in rodent and/or human
21.23%
21.52%
31.77%
31.63%
26.79%
26.50%
15.67%
16.54%
BL6
CBA
n= 683
log2(S-D1 over M-D1) in Human
c
Compartmental marker only in D2 either in rodent and/or human
21.90%
21.46%
29.20%
29.65%
24.34%
24.34%
21.02%
20.80%
BL6
CBA
n= 452
log2(S-D1 over M-D1) in Human
d
16.07%
17.29%
33.67%
33.06%
28.59%
27.57%
17.80%
18.62%
BL6
CBA
n= 983
log2(S-D2 over M-D2) in Rodent
log2(S-D2 over M-D2) in Human
Higher in S-D2
Higher in M-D2
e
21.52%
20.94%
31.33%
32.06%
25.18%
26.06%
17.42%
17.13%
BL2
CBA
n= 683
log2(S-D2 over M-D2) in Human
f
19.47%
20.35%
33.41%
32.30%
23.01%
21.68%
20.58%
21.90%
BL6
CBA
n= 452
log2(S-D2 over M-D2) in Human
g
D1/D2 marker in both compartments either in rodent and/or human
12.57%
13.36%
40.67%
41.26%
27.7%
27.31%
15.52%
14.73%
BL6
CBA
n= 509
log2(S-D2 over S-D1) in Rodent
log2(S-D2 over S-D1) in Human
Higher in S-D2
Higher in S-D1
h
D1/D2 marker only in striosome either in rodent and/or human
15.31%
15.14%
36.22%
34.86%
22.96%
23.64%
21.26%
22.79%
BL6
CBA
n= 588
log2(S-D2 over S-D1) in Human
i
D1/D2 marker only in matrix either in rodent and/or human
17.69%
17.35%
37.41%
34.69%
24.83%
25.17%
17.01%
19.73%
BL6
CBA
n= 294
log2(S-D2 over S-D1) in Human
j
11.79%
11.59%
40.86%
40.47%
28.29%
28.88%
15.52%
15.72%
BL6
CBA
n= 509
log2(M-D2 over M-D1) in Rodent
log2(M-D2 over M-D1) in Human
Higher in M-D2
Higher in M-D1
k
13.95%
14.63%
35.71%
35.37%
24.32%
24.15%
21.77%
22.28%
BL2
CBA
n= 588
log2(M-D2 over M-D1) in Human
l
13.95%
14.29%
38.1%
38.1%
27.21%
26.87%
17.69%
17.69%
BL6
CBA
n= 294
log2(M-D2 over M-D1) in Human

Supplementary Fig. 4 | The majority of S/M and D1/D2 marker genes are conserved across species, but a notable number of markers inverted their preference between rodent and human. a, For universal S/M marker genes found either in rodents or human, we plot log2(fold change) of their expression in S-D1 as compared to that in M-D1. Data for BL6 (blue) and CBA (green) controls are shown. Percentage in each quadrant indicates the proportion of genes falling in that quadrant. **b and c**, Same as **a**, but for compartmental markers found only in D1 (**b**) or D2 (**c**) population of either rodents or human. **d**, Same as **a**, but showing log2(fold change) of expressions in S-D2 as compared to M-D2. **e and f**, Same as **d**, but for compartmental markers found only in D1 (**e**) or D2 (**f**) population of either rodents or human. **g**, Same as **a**, but for universal D1/D2 marker genes found either in rodents or human. **h and i**, Same as **g**, but for D1/D2 markers found only in striosomes (**h**) or matrix (**i**) of either rodents or human. **j**, Same as **g**, but showing log2(fold change) of expressions between M-D1 and M-D2. **k and l**, Same as **j**, but for D1/D2 markers found only in striosomes (**k**) or matrix (**l**) of either rodents or human. We applied the criterion (abs(FC) > 0.1, $p < 0.001$) to define S/M markers that have a significant difference in the expression in striosomes and in matrix. Note that genes are included if they are listed as either of rodent (BL6 or CBA) or human markers. Thus, some genes appear in multiple panels; for example, KCNH7 is a universal striosome marker in rodents, whereas its differential expression across compartments was significant only in D1 population in human, appearing both in panels **a** and **b**. Differential gene expression analysis across cell-types, to define each marker, was performed using both the Wilcoxon rank-sum test and Welch's t test in order to independently confirm statistical results.

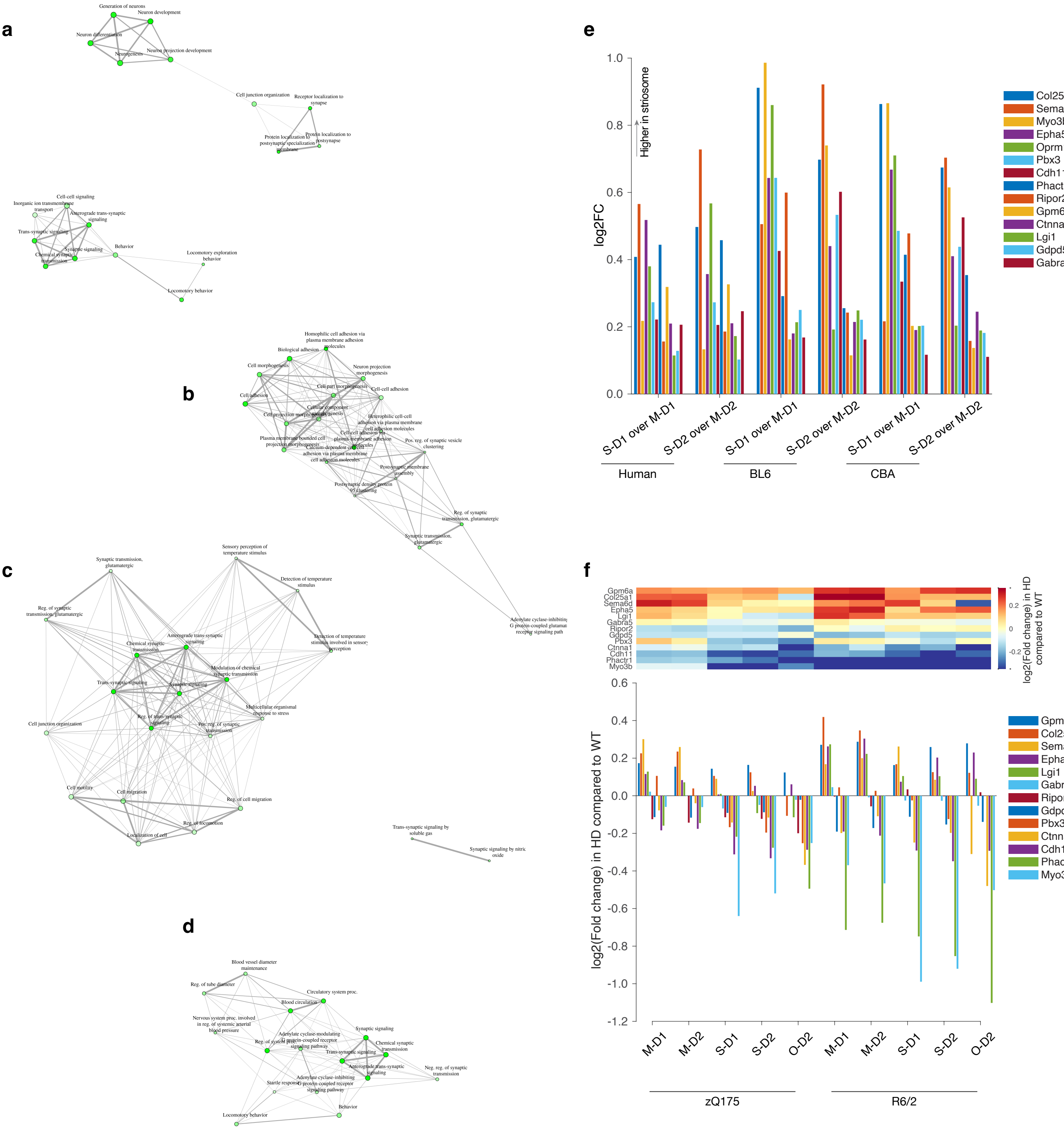

Supplementary Fig. 5 | In-depth analysis of hyper-conserved striosome markers involved in development. **a-d,** ShinyGO 0.76 (http://bioinformatics.sdstate.edu/go/) plots for hyper conserved striosome (**a**), matrix (**b**), D1 (**c**) and D2 (**d**) markers. **e,** Differential expression measured between the cell-type pairs indicated below and shown as log2(fold change) for human, BL6, and CBA dataset. We only included hyper-conserved striosome markers that are mapped onto GO terms related to development (i.e., cell morphogenesis involved in neuron differentiation, neuron development, neuron differentiation, generation of neurons, neurogenesis, and neuron projection development). **f,** Expressions in HD models compared to those in controls and shown as log2(fold change) using color code (top) and bars (bottom).

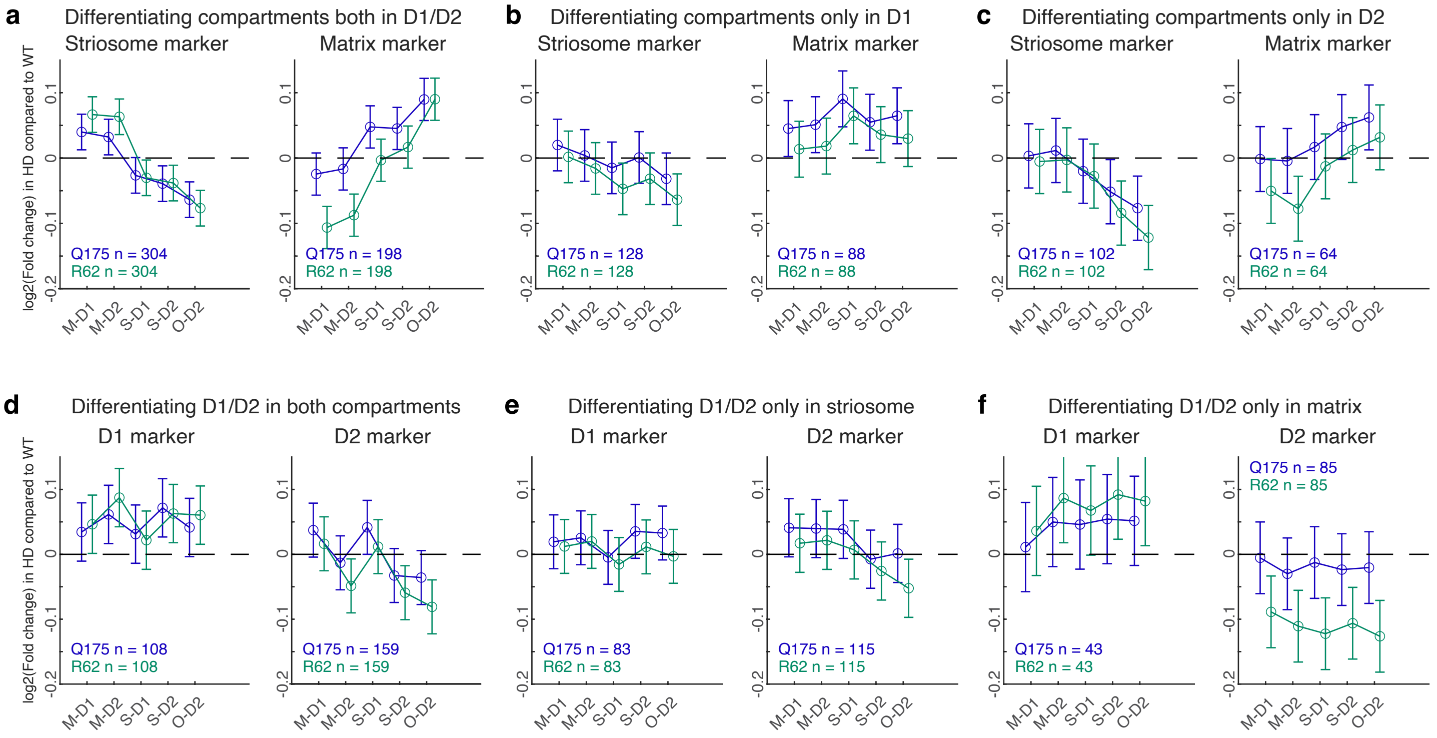

Supplementary Fig. 6 | Same analysis as in Figure 3 but including only strong, highly differentiating markers. We applied more strict criteria to define as marker (abs(log2FC) > 0.2) than those used in the analysis described in the main text (abs(log2FC) > 0.1). The criteria of FDR adjusted p values were the same, i.e., $p < 0.001$. **a**, Alteration of striosome (left) and matrix (right) marker expression, which is expressed more highly in striosomes or matrix both in D1/D2 populations, respectively, is shown for zQ175 (blue) and R6/2 (green) mice. N indicates number of markers included in each panel. Error bars indicate 95% confidence intervals of the averages. **b and c**, Same as in **a**, but for markers differentiating S/M only in dSPNs (**b**) or in iSPNs (**c**). **d**, Alteration of dSPN (left) and iSPN (right) marker expression, which is expressed more highly in D1 or D2 in both compartments, respectively, is shown for each model. N indicates number of markers included in each panel. Error bars indicate 95% confidence intervals of the averages. **e and f**, Same as in **d**, but for markers differentiating D1/D2 only in striosomes (**e**) or in matrix (**f**). Differential gene expression analysis across cell-types, to define each marker, was performed by both the Wilcoxon rank-sum test and Welch's t test in order to independently confirm statistical results.

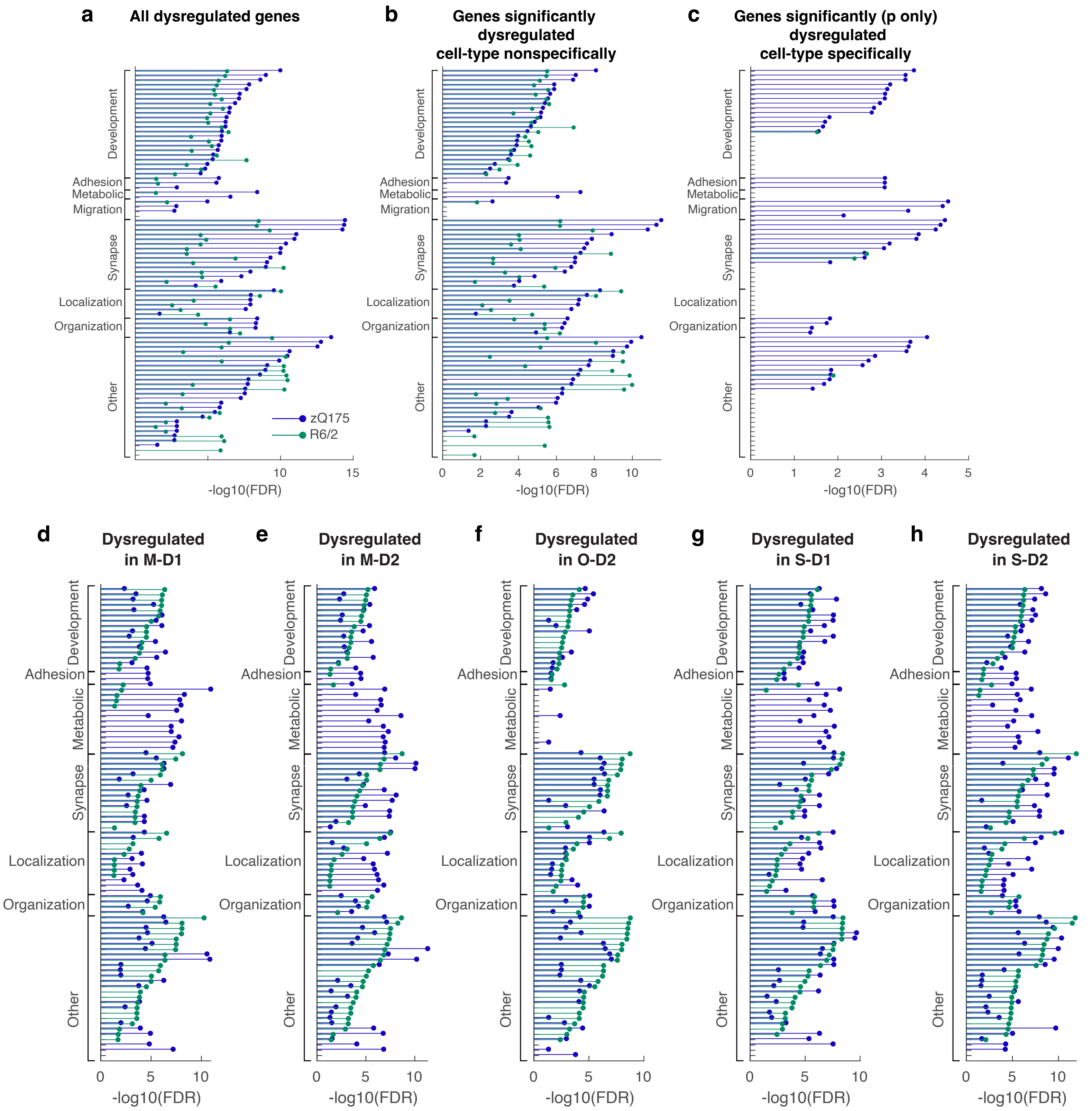

Supplementary Fig. 7 | GO analysis on dysregulated genes. a-c, FDRs for enriched GO terms are shown for all dysregulated genes detected with the criterion of abs(log2FC) > 0.1 and FDR-adjusted p value < 0.001 in at least one of four canonical cell types (**a**), or the subset of them that are unidirectionally dysregulated in all four canonical cell types (i.e., upregulation or downregulation in all four cell types, **b**). In **c**, we first selected genes with significant dysregulation (p < 0.001) in at least one of four canonical cell types, then further restricted to the genes that are dysregulated bidirectionally dependent on the cell types (i.e., upregulated in one cell type and downregulated in another cell type). **d-h**, FDRs for enriched GO terms are shown for all dysregulated genes detected in M-D1 (**d**), M-D2 (**e**), O-D2 (**f**), S-D1 (**g**), or S-D2 (**h**). Significance of dysregulations were statistically tested on a by-cell-type basis using both the Wilcoxon rank-sum test and Welch's t test in order independently to confirm statistical results. See Supplementary Table 5 for ID and full list of GO terms.

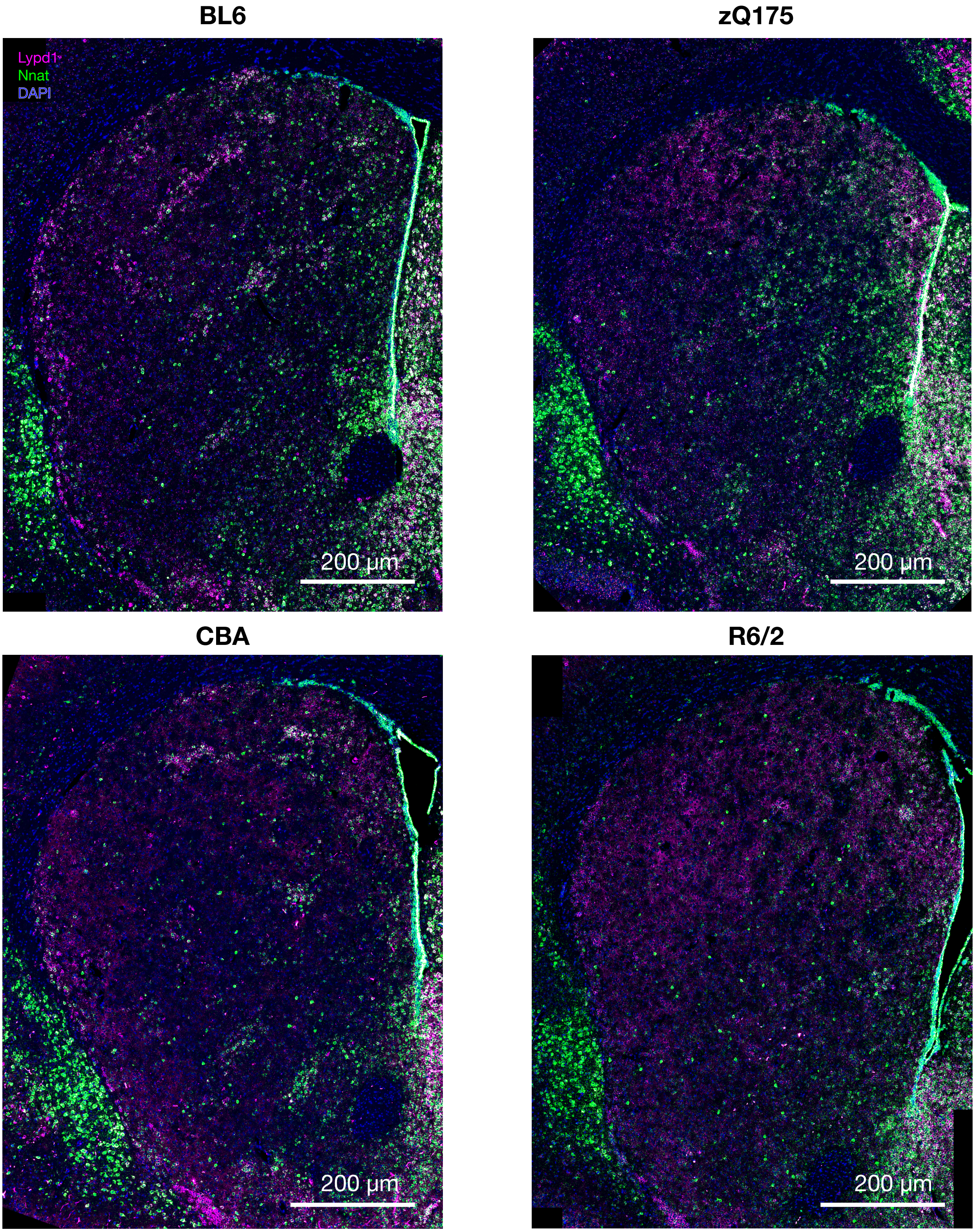

Supplementary Fig. 8 | Obscured compartmental organization in the two HD mouse models. Low magnification FISH images for the two HD models (left) compared to their controls (right), stained for striosome markers, i.e., Nnat(green), Lypd1(magenta), and DAPI (blue). Experiments are repeated in biologically independent animals (7 BL6 control, 6 zQ175, 8 CBA control and 8 R6/2 mice) to confirm similar results.
