# Supplementary material for: Transcriptional vulnerabilities of striatal neurons in human and rodent models of Huntington’s disease
Source: Nat Commun. 2023 Jan 17;14:282. doi: 10.1038/s41467-022-35752-x (PMC9845362; doi:10.1038/s41467-022-35752-x)
Supplement: Supplementary file 2 — Description of Additional Supplementary Files [file 41467_2022_35752_MOESM2_ESM.pdf]

Description of Additional Supplementary Files

File Name: Supplementary Dataset 1.

Description: List of samples used in this study, the curated set of compartmental markers, and list of O-D2 markers, related to Figure 1. We searched for genes whose expression in O-D2 is highly enriched as compared to the ground average expression level in the striatum (including non-SPNS), i.e., abs(log2FC) > 0.1; p < 1e-05, and not enriched in any other cell types, i.e., abs(log2FC) < 0.1; p > 0.001. Differential gene expression analysis of the snRNA-seq data was performed on a by-cell-type basis by both the Wilcoxon rank-sum test and Welch's t test, using both limma and DESeq2, in order to independently confirm statistical results.

File Name: Supplementary Dataset 2.

Description: List of GO terms enriched in universal striosome, matrix, D1, or D2 markers compared across human, BL6, and CBA samples, list of GO terms in the order shown in Figure 1E-H, and list of GO terms in the order shown in Figure 4K-N, related to Figures 1 and 4. Differential gene expression analysis of the snRNA-seq data was performed on a by-cell-type basis by both the Wilcoxon rank-sum test and Welch's t test, using both limma and DESeq2, in order to independently confirm statistical results. All GO analysis used the PANTHER overrepresentation test (Released 20210224)[65] and Gene Ontology database DOI:10.5281/zenodo.5228828 Released 2021-08-18, FISHER test (http://geneontology.org/).

File Name: Supplementary Dataset 3.

Description: Matrix of Jensen-Shannon distance between SPN subtypes, and matrix of Jensen-Shannon distance between all striatal subtypes, related to Figure 2.

File Name: Supplementary Dataset 4.

Description: Dysregulation of all significantly dysregulated genes (i.e., including non-markers), significantly dysregulated striosome or matrix markers, and significantly dysregulated D1 or D2 markers are shown in separate sheet for each of R6/2 and zQ175 models, related to Figure 4. Within each sheet, genes dysregulated in a cell-type nonspecific manner (i.e., upregulated in all 4 canonical SPN subtypes, or downregulated in all 4 canonical SPN subtypes, with criteria abs(log2(fold change)) > 0.1 and FDR-adjusted p < 0.001), or specific manner (i.e., upregulated in one subtype while downregulated in other(s), with criteria FDR-adjusted p < 0.001) are listed in leftward columns as well. Differential gene expression analysis of the snRNA-seq data was performed on a by-cell-type basis by both the Wilcoxon rank-sum test and Welch's t test, using both limma and DESeq2, in order to independently confirm statistical results.

File Name: Supplementary Dataset 5.
Description: List of GO terms enriched in genes dysregulated significantly at least in one cell-type (Any), cell-type-nonspecifically dysregulated genes (NONspe), and cell-type-specifically dysregulated genes (Spe), separately for zQ175 and R6/2 model, related to Supplementary Fig. 8. For the categorization, see the main text. Differential gene expression analysis of the snRNA-seq data was performed on a by-cell-type basis by both the Wilcoxon rank-sum test and Welch's t test, using both limma and DESeq2, in order to independently confirm statistical results. All GO analysis used the PANTHER overrepresentation test (Released 20210224)[65] and Gene Ontology database DOI:10.5281/zenodo.5228828 Released 2021-08-18, FISHER test (http://geneontology.org/).

File Name: Supplementary Dataset 6.
Description: Statistical results applied to firing frequency curves shown in Figure 6I-N
