# Supplementary material for: Transcriptional vulnerabilities of striatal neurons in human and rodent models of Huntington’s disease
Source: Nat Commun. 2023 Jan 17;14:282. doi: 10.1038/s41467-022-35752-x (PMC9845362; doi:10.1038/s41467-022-35752-x)
Supplement: Supplementary file 9 — Reporting Summary [file 41467_2022_35752_MOESM9_ESM.pdf]

Corresponding author(s): Ann Graybiel, Myriam Heiman, Manolis Kellis

Last updated by author(s): Sep 7, 2022

Reporting Summary

Nature Portfolio wishes to improve the reproducibility of the work that we publish. This form provides structure for consistency and transparency in reporting. For further information on Nature Portfolio policies, see our Editorial Policies and the Editorial Policy Checklist.

Statistics

For all statistical analyses, confirm that the following items are present in the figure legend, table legend, main text, or Methods section.

n/a	Confirmed	
☐	☒	The exact sample size (n) for each experimental group/condition, given as a discrete number and unit of measurement
☐	☒	A statement on whether measurements were taken from distinct samples or whether the same sample was measured repeatedly
☐	☒	The statistical test(s) used AND whether they are one- or two-sided<br>*Only common tests should be described solely by name; describe more complex techniques in the Methods section.*
☒	☐	A description of all covariates tested
☐	☒	A description of any assumptions or corrections, such as tests of normality and adjustment for multiple comparisons
☐	☒	A full description of the statistical parameters including central tendency (e.g. means) or other basic estimates (e.g. regression coefficient) AND variation (e.g. standard deviation) or associated estimates of uncertainty (e.g. confidence intervals)
☐	☒	For null hypothesis testing, the test statistic (e.g. F, t, r) with confidence intervals, effect sizes, degrees of freedom and P value noted<br>*Give P values as exact values whenever suitable.*
☒	☐	For Bayesian analysis, information on the choice of priors and Markov chain Monte Carlo settings
☒	☐	For hierarchical and complex designs, identification of the appropriate level for tests and full reporting of outcomes
☒	☐	Estimates of effect sizes (e.g. Cohen's d, Pearson's r), indicating how they were calculated

Our web collection on statistics for biologists contains articles on many of the points above.

Software and code

Policy information about availability of computer code

Data collection	pCLAMP 11: Electrophysiology data acquisition.<br>Details for RNA-seq are included in the methods.
Data analysis	Cellranger v4.0: Read alignment and count matrix generation.<br>ACTIONet v3.0.0, scran v1.18.5: Count preprocessing, quality control, clustering, and annotation. limma v3.46.0, DESeq2 v1.30.1: Differential expression analysis.<br>PANTHER : GO analysis.<br>GraphPad Prism Version 9.3.1 : statistical analysis on firing frequency curves.<br>R v4.1: Standard statistical analysis.<br>MATLAB R2021a, R2022a: Standard statistical analysis.

For manuscripts utilizing custom algorithms or software that are central to the research but not yet described in published literature, software must be made available to editors and reviewers. We strongly encourage code deposition in a community repository (e.g. GitHub). See the Nature Portfolio guidelines for submitting code & software for further information.

Data

Policy information about availability of data

All manuscripts must include a data availability statement. This statement should provide the following information, where applicable:

- Accession codes, unique identifiers, or web links for publicly available datasets
- A description of any restrictions on data availability
- For clinical datasets or third party data, please ensure that the statement adheres to our policy

Raw and processed sequencing data, including annotated count matrices, are publicly available in NCBI GEO under accession # GEO: GSE152058.
Gene Ontology database DOI:10.5281/zenodo.5228828 Released 2021-08-18, FISHER test (http://geneontology.org/)

Human research participants

Policy information about studies involving human research participants and Sex and Gender in Research.

Reporting on sex and gender	N/A
Population characteristics	N/A
Recruitment	N/A
Ethics oversight	Human tissue analyses were conducted as exempt human research, as this was secondary research using bio-specimens not specifically collected for this study. All samples were obtained from biobanks/repositories using appropriate de-identification and under consent.

Note that full information on the approval of the study protocol must also be provided in the manuscript.

Field-specific reporting

Please select the one below that is the best fit for your research. If you are not sure, read the appropriate sections before making your selection.

☒ Life sciences ☐ Behavioural & social sciences ☐ Ecological, evolutionary & environmental sciences

For a reference copy of the document with all sections, see nature.com/documents/nr-reporting-summary-flat.pdf

Life sciences study design

All studies must disclose on these points even when the disclosure is negative.

Sample size	Sample size for mouse studies was determined based on sufficient statistical power obtained in the similar prior studies (Lee et al., 2020; Langfelder, P. et al. 2016). Samples sizes of human were restrained by the sample availability of the rare and precious grade 1 HD post-mortem brains, but whenever possible, set to be >3.
Data exclusions	No data were excluded from analysis.
Replication	Validation of sequencing results was performed by fluorescent in situ hybridization using probes targeting the computationally detected transcripts. We have tested at least 10 probes to find consistent results with the transcriptional dataset. Also, we conducted electrophysiological experiments to confirm computationally predicted outcomes of disease-related transcriptional dysregulations. Recordings were made in total from 212 SPNs in 10 control mice and from 198 SPNs in 9 heterozygous mice. The mean ± SD number of cells recorded per each mouse evaluated was 22 ± 6. Approximately equal numbers of putative striosome and matrix SPNs were recorded per mouse.
Randomization	Randomization was not necessary in this study given the unbiased experimental approach. Covariates were randomized during differential expression analysis for permutation testing to assure that differential expression results were not driven by random variation.
Blinding	Blinding is not relevant to this study as no qualitative analyses were employed.

Reporting for specific materials, systems and methods

We require information from authors about some types of materials, experimental systems and methods used in many studies. Here, indicate whether each material, system or method listed is relevant to your study. If you are not sure if a list item applies to your research, read the appropriate section before selecting a response.

Materials & experimental systems

n/a	Involved in the study	
☒	☐	Antibodies
☒	☐	Eukaryotic cell lines
☒	☐	Palaeontology and archaeology
☐	☒	Animals and other organisms
☒	☐	Clinical data
☒	☐	Dual use research of concern

Methods

n/a	Involved in the study	
☒	☐	ChIP-seq
☒	☐	Flow cytometry
☒	☐	MRI-based neuroimaging

Animals and other research organisms

Policy information about studies involving animals; ARRIVE guidelines recommended for reporting animal research, and Sex and Gender in Research

Laboratory animals	Mouse B6CBA-Tg(HDexon1)62Gpb/1J mice (CAG repeat length 160 ± 5; Jackson Laboratories stock # 002810) at 9 weeks of age.<br>Mouse B6J.zQ175DN (Jackson Laboratories stock # 370832) at 6 months of age.<br>Mouse: R6/2 non-carrier: B6CBA-Tg(HDexon1) 62Gpb/1J non carrier controls at 9 weeks of age.<br>Mouse: C57BL/6J WT controls at 6 months of age.
Wild animals	No wild animals were used in the study.
Reporting on sex	All mouse studies employed only male mice due to the well-documented high, non-disease-associated variability observed in females of the mouse models used.
Field-collected samples	No field-collected samples were used in this study.
Ethics oversight	All mouse husbandry and experimental procedures were conducted with the approval of the Committee on Animal Care at the Massachusetts Institute of Technology.

Note that full information on the approval of the study protocol must also be provided in the manuscript.
